# A National HIV Provider Survey of Antiretroviral Therapy Preferences for Management of Treatment-Naive and Experienced Individuals With Drug Resistance

**DOI:** 10.1093/ofid/ofad541

**Published:** 2023-10-31

**Authors:** Sonya Krishnan, Christopher K Lippincott, Stephanie Bjerrum, Marina B Martinez Rivera, Maunank Shah

**Affiliations:** Division of Infectious Diseases, Department of Medicine, School of Medicine, Johns Hopkins University, Baltimore, Maryland, USA; Division of Infectious Diseases, Department of Medicine, School of Medicine, Johns Hopkins University, Baltimore, Maryland, USA; Department of Infectious Diseases, Copenhagen University Hospital–Rigshospitalet, Copenhagen, Denmark; Division of Infectious Diseases, Department of Medicine, School of Medicine, Johns Hopkins University, Baltimore, Maryland, USA; Division of Infectious Diseases, Department of Medicine, School of Medicine, Johns Hopkins University, Baltimore, Maryland, USA

**Keywords:** HIV drug resistance, HIV guidelines, HIV provider, survey

## Abstract

**Background:**

HIV clinical practice guidelines outline broad treatment principles but offer less explicit recommendations by permutations of encountered viral resistance. We hypothesize that there is variability in antiretroviral (ARV) regimen decision making among providers when considering HIV drug resistance (HIVDR).

**Methods:**

US HIV providers provided ARV regimen recommendations for case vignettes in a series of electronic surveys encompassing variations of HIVDR. Responses were characterized by drugs and classes selected and anticipated activity based on genotypic susceptibility. Heterogeneity was defined as the proportion of unique ARV regimens from total responses.

**Results:**

An overall 119 providers from the United States participated. Among case vignettes with isolated M184V and viremia, 85.9% selected a regimen with 2 nucleoside reverse transcriptase inhibitors (NRTIs) + integrase strand transfer inhibitor (INSTI); 9.9% selected regimens with >3 ARVs. Alternatively, in scenarios of viremia with moderate to high-level NRTI resistance, >50% of providers selected an NRTI-sparing regimen, while a minority recommended 2 NRTIs + INSTI (21/123, 17%). In moderate to high-level INSTI resistance, there was response heterogeneity, with no common unifying approach to management (127 unique regimens/181 responses, 70% heterogeneity). Providers used cabotegravir/rilpivirine for treatment simplification in suppressed cases, despite a history of treatment failure (37/205, 36%).

**Conclusions:**

Our national survey of US HIV providers revealed a consensus to management of HIV resistance with potential alternative options in cases with low heterogeneity. Providers selected cabotegravir/rilpivirine as a viable treatment simplification strategy in suppressed cases with a history of treatment failure. The responses to the case vignettes could be used an education tool for ARV decision making in HIVDR.

Despite an expanding list of antiretrovirals (ARVs), HIV drug resistance (HIVDR) remains problematic. Pretreatment, transmitted resistance can exceed 15% in some global settings, with estimates exceeding 20% with prior drug exposure [1]. Additionally, people with HIV continue to develop acquired ARV resistance while undergoing therapy, with failure of nonnucleoside reverse transcriptase inhibitor (NNRTI)–anchored treatment leading to resistance mutations to commonly used nucleoside reverse transcriptase inhibitors (NRTIs), such as M184V mutations and higher-level NRTI resistance mutations, such as K65R and thymidine analog mutations (TAMs) [1–3]. Despite development of high-barrier-to-resistance integrase strand transfer inhibitors (INSTI), such as dolutegravir (DTG) and bictegravir (BIC), resistance in clinical trials and real-world practice has emerged [4–8].

Although the widespread prevalence of HIVDR exists, there is clinical equipoise on the best ARV regimen for management in specific situations. For example, guidelines offer general management strategies for virologic failure, such as the importance of adherence counseling, a recommendation against the addition of a single ARV to a failing regimen, and direction that a regimen include 2 fully active ARVs if 1 of the drugs has a high barrier to resistance but outline that 3 fully active drugs are preferred [9, 10]. Thus, current guidelines offer treatment principles rather than dictate recommendations for specific resistance patterns encountered in practice. Additionally, guidelines may not always reflect rapidly emerging literature. Consequently, there is variability in decision making among HIV providers in the setting of HIVDR.

To date, clinical practice heterogeneity among HIV providers in HIVDR has not been well categorized. We therefore sought to ascertain provider practices for hypothetical case scenarios involving patients who were treatment naive and experienced viremia and suppression with varying degrees of drug resistance mutations. In particular, we sought to identify patterns in ARV regimen construction in the setting of NRTI resistance with an M184V alone vs (1) more extensive NRTI resistance (eg, K65R, TAMs), (2) INSTI resistance mutations, or (3) other multiclass resistance and to evaluate usage of cabotegravir/rilpivirine (CAB/RPV).

## METHODS

### Participant Selection

From 26 August 2022 to 11 December 2022, clinicians were emailed a Qualtrics e-survey through academic institutions and multiple professional society networks, in an attempt to identify HIV providers across the continental United States. These individuals were invited to disseminate the invitation to other HIV providers, such as attending physicians, pharmacists, and advanced practice providers within their institutes, clinics, and professional networks.

### Ethics

This study was approved by the Johns Hopkins University Institutional Review Board. All participants consented to participate, affirming their consent through a survey question.

### Standardized Case Scenarios

We developed hypothetical standardized clinical case vignettes, stratified by scenarios involving patients who were treatment naive, treatment experienced and suppressed, or treatment experienced and viremic; cases contained varying degrees of HIV resistance and other modifying factors. Analysis included all participants who enrolled in the study and completed an initial survey of 6 cases, which comprised a random selection with and without HIVDR. Participants had the option to complete additional surveys, with a total of 36 cases possibly completed per participant. In this article, we present results from 16 cases involving drug resistance. The full clinical vignettes are available in Supplementary Table 2. To facilitate study participation, these scenarios were divided into blocks of 6 cases, which participants were randomly assigned in each survey, with the option to complete additional blocks. Cases were grouped into those involving M184V alone, extensive NRTI resistance (including tenofovir [TXF; K65R or TAMs]), INSTI resistance mutations, and other multiclass resistance.

### Data Collection and Statistical Analyses

We conducted descriptive statistics (Stata version 17.0; StataCorp) to describe participant demographics and the frequency of regimen selections per case vignette. Participant responses were excluded if incomplete (eg, blank, monotherapy). For each case scenario, we described the ARV regimen responses, the calculated genotypic susceptibility score (GSS) of the selected regimens, and whether NRTIs and INSTIs were included in the regimen. The GSS for each participant-selected regimen was based on the Stanford HIV Drug Resistance Database (Stanford HDRD) scoring system, defined as follows: < 10, susceptible; 10 to 14, potential low-level resistance; 15 to 29, low-level resistance; 30 to 59, intermediate resistance; ≥60, high-level resistance [11, 12]. Similar to prior studies, the predicted activity of a drug was scaled from 0 to 1, with 1 representing a fully active drug; drugs with low-level, moderate, or high-level resistance were assigned GSSs of 0.66, 0.33, and 0, respectively [13, 14].

In the absence of a universally accepted measure of survey response heterogeneity, we report heterogeneity as the number and proportion of unique ARV regimens recommended by participants, calculated as follows: (unique ARV regimens/total responses) × 100, where 100% represents complete heterogeneity in which every participant suggested a different ARV regimen. A lower percentage indicates less heterogeneity. A unique regimen was defined by drug (not drug class).

## RESULTS

### Participant Characteristics

**Figure 1.**
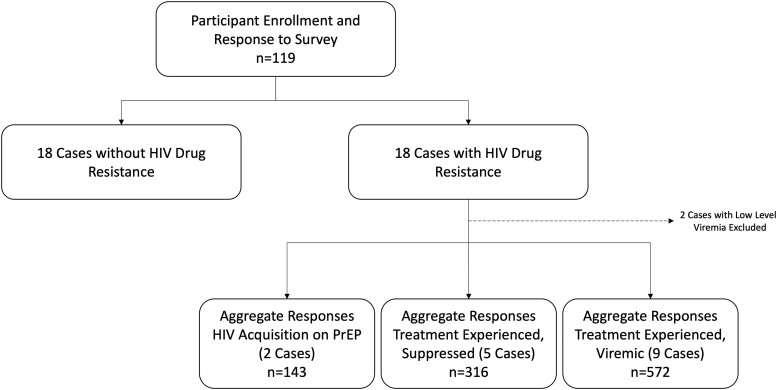
Study flowchart. Only providers (MD/DO, advanced practice provider, pharmacists) who reported experience proving care for people living with HIV were included in this study. Individuals were invited to participate and complete the survey. Among those who completed survey 1, a subsequent invite was sent for survey 2 and then survey 3. Each survey contained 2 blocks of 6 cases comprising a mixture of those with and without HIV resistance. PrEP, pre-exposure prophylaxis.

An overall 119 providers participated in this survey (Figure 1). Data on 16 cases involving drug resistance were included in the current analysis (median, 65 responses per case). Respondents were mostly physicians (n = 87, 73.1%) from an infectious diseases specialty (n = 98, 82.4%) and practicing in an academic/university setting (n = 108, 90.8%; Table 1). Most frequently, respondents had ≥10 years of clinical experience (n = 51, 42.9%), and 65.5% (n = 78) spent >25% of their clinical time caring for people with HIV, with all regions of the continental United States represented.

**Table 1.** Characteristics of Survey Respondents (N = 119)

Characteristic	No. (%)
Clinical role	
Physician	87 (73.1)
Advanced practice provider	17 (14.3)
Pharmacist	14 (11.8)
Other	1 (0.8)
Specialty	
Infectious diseases	98 (82.4)
Internal medicine	10 (8.4)
General practice/family medicine	8 (6.7)
Other	3 (2.5)
Geographic location	
Mid-Atlantic	32 (26.9)
Midwest	30 (25.2)
West	19 (16.0)
Southeast	15 (12.6)
Northwest	10 (8.4)
Southwest	9 (7.6)
Northeast	4 (3.4)
Alaska and Pacific Islands/other US territory	0 (0)
Practice setting	
Academic/university	108 (90.8)
FQHC/CHC	4 (3.4)
Health department/government	4 (3.4)
Private practice	1 (0.8)
HMO/network	1 (0.8)
Other	1 (0.8)
Clinical experience, y	
≤1	4 (3.4)
2–4	37 (31.1)
5–7	20 (16.8)
7–9	7 (5.9)
≥10	51 (42.9)
Clinical time caring for PWH, %	
<25	41 (34.5)
25–50	37 (31.1)
51–75	11 (9.2)
>75	30 (25.2)

Abbreviations: FQHC/CHC, federally qualified health center/community health center; HMO, health maintenance organization; PWH, people with HIV.

#### Isolated M184V Resistance Without INSTI Resistance

##### Viremic Cases

We included 2 case scenarios with detectable viral load and confirmed or suspected M184V transmitted resistance (Table 2, cases V1 and V2). In case V1 with a confirmed M184V, among 71 respondents there was relatively little heterogeneity (15.5%) with 11 unique ARV regimens indicated (Table 3). Overall, 85.9% (61/71) suggested pairing 2 NRTIs with a second-generation INSTI (n = 55, 77.5%; BIC/TAF/FTC), while 7 (9.9%) chose an intensified regimen with >3 drugs. In case V2 (pending baseline resistance), there were 10 unique ARV regimens offered (13.9% heterogeneity). In contrast to case V1, a larger proportion recommended pairing a protease inhibitor (PI; boosted DRV [DRV/b]) with 2 NRTIs (12.5%, case V2; 1.4%, case V1) or considered an intensified regimen with >3 drugs (15.3%, case V2; 9.9%, case V1).

**Table 2.** Cases Involving Drug Resistance and Detectable Viremia

			Most Common Responses	
Case	Case Summary^a^	Cumulative Resistance	Class^b,c^	No. (%)
V1	23 y, F VL, 125 K CD4 >200 New diagnosis with intermittent PrEP adherence (TDF/FTC)	NRTI: M184V	1 INSTI, 2 NRTI 1 PI, 1 INSTI, 2 NRTI Other	61 (85.9) 7 (9.9) 3 (4.2)
V2	53 y, M VL, 400 K New diagnosis with intermittent PrEP adherence (TDF/FTC)	Pending	1 INSTI, 2 NRTI 1 PI, 1 INSTI, 2 NRTI 1 PI, 2 NRTI 1 PI, 1 INSTI	51 (70.8) 11 (15.3) 9 (12.5) 1 (1.4)
V3	36 y, M VL, 170 K CD4, 210 Currently taking EFV/TDF/FTC with intermittent adherence	NRTI: M184V, K65R, Y115F NNRTI: K103N	1 PI, 1 INSTI, 1 NNRTI 1 INSTI, 2 NRTI 1 PI, 1 INSTI Other	12 (22.6) 11 (20.8) 11 (20.8) 19 (35.8)
V4	34 y, M Distant diagnosis CD4, 95 VL, 110 K Extensive treatment experience and reengaging in care, desires few pills	NRTI: M184V, M41L, T215Y, L210W	1 PI, 1 INSTI 1 INSTI, 2 NRTI 1 PI, 1 INSTI, 2 NRTI 1 INSTI, 1 NNRTI Other	18 (25.7) 10 (14.3) 10 (14.3) 9 (12.9) 23 (32.8)
V5	37 y, M VL, 55 K CD4, 184 Prior exposure to NRTIs, PIs, and NNRTIs	NRTI: M184V, M41L, D67N, L74V, L210W, T215D, K219N NNRTI: K103N PI: L90M INSTI: Y143C, T97A	1 PI, 1 INSTI, 2 NRTI 1 PI, 1 INSTI 1 PI, 1 INSTI, 1 NNRTI Other	17 (25.0) 12 (17.6) 12 (17.6) 27 (39.7)
V6	48 y, M VL, 310 K CD4 <200 Taking EVG/c/TAF/FTC with intermittent adherence	NRTI: M184V INSTI: E92Q	1 PI, 2 NRTI 1 PI, 1 INSTI, 2 NRTI 1 INSTI, 2 NRTI Other	24 (31.2) 22 (28.6) 18 (23.4) 13 (16.9)
V7	60 y, M VL, 12 K CD4 196 Taking DRV/r BID, RAL, TDF/FTC	NRTI: M41L, D67N, T69A, K70R, L74V, M184V, T215Y, K219Q NNRTI: K101P, K103N PI: I54V, I84V INSTI: E92Q	1 PI, 1 INSTI, 1 EI 1 PI, 1 INSTI, 1 NNRTI 1 PI, 1 INSTI, 2 NRTI Other	9 (14.5) 9 (14.5) 7 (11.3) 37 (59.7)
V8	44 y, M VL, 185 K Unknown prior regimen, viremic with BIC/TAF/FTC	NRTI: D67N, K70R, M184V, K65R NNRTI: K101E, V108I PI: L33F, I50V, I54L INSTI: T97A, N155H	1 INSTI, 1 EI, 1 NNRTI 1 PI, 1 INSTI, 1 NNRTI Other	9 (14.8) 8 (13.1) 44 (72.1)
V9	46 y, F VL, 430 K CD4 <50 Taking ETR, DTG, DRV/r, TAF/FTC Prior treatment failure with EFV/TDF/FTC and RAL + TDF/FTC	NRTI: M41L, D67N, T69D, K70Q, M184V, T215F, L74I NNRTI: Y181C, K103N PI: V32I, L33F, M46L, I54V, I84V, L90M INSTI: G140S, Q148H	1 PI, 1 INSTI, 2 NRTI, 1 NNRTI 1 PI, 1 INSTI, 1 EI, 2 NRTI, 1 NNRTI 1 PI, 1 INSTI, 1 EI Other	6 (8.2) 5 (6.8) 4 (5.5) 58 (79.4)
V10	28 y, F With perinatally acquired HIV VL, 10–15 K CD4, 180 Taking DTG + RPV/TAF/FTC with preference for few pills	NRTI: M41L, M184V, T215Y NNRTI: K101P, V106I, V179F, Y181C PI: V82A, L90M, K20T INSTI: E92Q, G140S, Q148R Other: T69I	1 PI, 1 EI, 2 NRTI 1 PI, 2 NRTI 1 PI, 1 INSTI, 2 NRTI Other	8 (15.4) 6 (11.5) 5 (9.6) 33 (63.5)
V11	49 y, M VL, 32.5 K CD4, 38 Detectable viremia on DRV/r BID + DTG BID + TDF/FTC Extensive prior antiretroviral exposure to NRTIs, NNRTIs, PIs, and INSTIs	NRTI: M41L, E44A, T74P, V75M, F77L, M184V, L210W, T215Y NNRTI: K103N, G190A PI: V32I, L33F, I54L, I84V INSTI: G140S, Q148H Other: V11I, V118V/I	1 INSTI, 1 EI, 1 NNRTI 1 INSTI, 2 EI, 1 NNRTI 1 PI, 1 INSTI, 1 EI 1 INSTI, 1 EI, 2 NRTI, 1 NNRTI Other	8 (14.3) 7 (12.5) 5 (8.9) 4 (7.1) 32 (57.1)

Abbreviations: EI, entry inhibitor; K, thousand; INSTI, integrase strand transfer inhibitor; NNRTI: nonnucleoside reverse transcriptase inhibitor; NRTI, nucleoside reverse transcriptase inhibitor; PI, protease inhibitor; PrEP, pre-exposure prophylaxis; VL, viral load.

^a^For a list of antiretroviral abbreviations, full clinical vignette details, as well as specific antiretroviral regimen responses, see Supplementary Tables 1–3.

^b^Second-generation INSTIs were selected by providers (ie, dolutegravir, bictegravir, cabotegravir); no first-generation INSTIs were selected (ie, elvitegravir and raltegravir); boosted darunavir was selected in all cases as the PI, with the exception of case V8, where 4 selected boosted atazanavir and 26 selected boosted darunavir.

^c^Cabotegravir/rilpivirine use as follows: V3, n = 1 (1.9%); V4, n = 6 (8.6%); V5, n = 2 (2.9%); V6, n = 4 (5.2%); V8, n = 1 (1.6%), V9, n = 1 (1.4%), V11, n = 2 (3.6%).

**Table 3.** Comparison of Low to Moderate/High Level of NRTI Resistance Treatment Responses, Without Concomitant PI or INSTI Resistance

		No. (%)							
NRTI Resistance^a^: Case	No.	2 NRTI + INSTI	2-Drug Regimen	2 NRTI + PI	NRTI Sparing	EI + OBR	>3-Drug Regimen	Heterogeneity,^b^ % (No.)	GSS,^c^ Median (IQR)
Isolated M14V, No Tenofovir Resistance									
Viremic									
V1	71	61 (85.9)	1 (1.4)	1 (1.4)	1 (1.4)	0	7 (9.9)	15.5 (11/71)	2.33 (2.33–2.33)
V2	72	51 (70.8)	1 (1.4)	9 (12.5)	1 (1.4)	0	11 (15.3)	13.9 (10/72)	Unknown
Suppressed									
S1	77	42 (54.5)	20 (26.0)	12 (15.6)	21 (27.3)	0	10 (13.0)	11.7 (9/77)	2.33 (2–2.33)
S2	71	56 (78.9)	13 (18.3)	1 (1.4)	13 (18.3)	0	0	11.3 (8/71)	2.33 (2.33–2.33)
S3	57	42 (73.7)	15 (26.3)	0	14 (24.6)	0	0	8.8 (5/57)	2.33 (2–2.33)
M184V + Tenofovir Resistance									
Viremic									
V3	53	11 (20.8)	16 (30.2)	1 (1.9)	28 (52.8)	0	9 (17.0)	39.6 (21/53)	2 (2–2)
V4	70	10 (14.3)	28 (40.0)	1 (1.4)	35 (50.0)	1 (1.4)	20 (28.6)	34.3 (24/70)	2 (2–2.33)
Suppressed									
S4	54	28 (51.9)	21 (38.9)	1 (1.9)	20 (37.0)	0	3 (5.6)	26.0 (14/54)	1.33 (1.33–2)
S5	57	8 (14.0)	39 (68.4)	7 (12.3)	37 (64.9)	0	3 (5.3)	14.0 (8/57)	2 (1.33–2)

Abbreviations: EI, entry inhibitor; GSS, genotypic susceptibility score; INSTI, integrase strand transfer inhibitor; NRTI, nucleoside reverse transcriptase inhibitor; PI, protease inhibitor; OBR, optimized background regimen.

^a^Case scenarios did not have concomitant PI or INSTI resistance.

^b^Heterogeneity was defined as follows: (unique antiretroviral regimens / total responses) × 100, where 100% represents complete heterogeneity in which every participant suggested a different antiretroviral regimen. A lower percentage indicates less heterogeneity. A unique regimen was defined by drug (not drug class).

^c^GSS represents a sum of each regimen's expected antiretroviral activity. Each drug was scaled from 0 to 1, with 1 representing a fully active drug. Drugs with low-level, intermediate, or high-level resistance (based on Stanford HIV Drug Resistance Database) are assigned GSSs of 0.66, 0.33, and 0, respectively. The Stanford HIV Drug Resistance Database assigns scores as follows: <10, susceptible; 10–14, potential low-level resistance; 15–29, low-level resistance; 30–59, intermediate resistance; ≥60, high-level resistance.

##### Suppressed Cases

We explored ARV selection among 3 scenarios with suppressed viral load but a history of NNRTI-anchored treatment failure. We evaluated ARV regimen optimization on an intensified regimen (TAF/FTC/DRV/c + DTG; underlying M184V) in an individual desiring simplification (case S1). Among 77 responses (11.7% heterogeneity), the majority suggested simplification to BIC/TAF/FTC (n = 41, 53.3%), while 21 (27.3%) cited an NRTI-sparing regimen with CAB/RPV (n = 11, 14%) or DTG/RPV (n = 9, 11.7%; Tables 3 and 4, Supplementary Table 3).

**Table 4.** Cases Involving Drug Resistance and Viral Suppression

			Most Common Responses	
Case^a^	Case Summary	Cumulative Resistance	Classes^b,c^	No. (%)
S1	43 y, M VL <20 CD4 >200 Taking TAF/FTC/DRV/c + DTG Prior treatment failure (unknown regimen) Requests simplification	NRTI: M184V NNRTI: K103N	1 INSTI, 2 NRTI 1 INSTI, 1 NNRTI 1 PI, 1 INSTI, 2 NRTI Other	42 (54.5) 20 (26.0) 9 (11.7) 6 (7.8)
S2	37 y, M VL <20 CD4, 375 Taking EVG/c/TDF/FTC Prior failure of EFV/TDF/FTC	NRTI: M184V	1 INSTI, 2 NRTI 1 INSTI, 1 NNRTI Other	56 (78.9) 13 (18.3) 2 (2.8)
S3	24 y, F VL <20 CD4 >200 Taking BIC/TAF/FTC Perinatal HIV, desires few pills Prior AZT, AZT/ABC/3TC, LPV/r + AZT/3TC, EFV/TDF/FTC, ATV/r + TDF/FTC, suppressed >1 y	NRTI: M184V	1 INSTI, 2 NRTI 1 INSTI, 1 NNRTI 1 INSTI, 1 NRTI	42 (73.7) 14 (24.6) 1 (1.7)
S4	64 y, F VL <20 CD4, 380 Taking DTG + TDF/FTC Prior EFV/TDF/FTC	Archived genotype NRTI: K65R, M184V NNRTI: K103N	1 INSTI, 2 NRTI 1 INSTI, 1 NNRTI 1 PI, 1 INSTI Other	28 (51.8) 17 (31.5) 3 (5.6) 6 (11.1)
S5	36 y, F VL <20 CD4, 440 Taking DRV/r + TDF/FTC for 12 mo Prior EFV/TDF/FTC Preferences for decreased pill burden	NRTI: M184V, M41L, T215Y, L210W	1 INSTI, 1 NNRTI 1 INSTI, 2 NRTI 1 PI, 2 NRTI Other	37 (64.9) 8 (14.0) 7 (12.3) 5 (8.8)

Abbreviations: INSTI, integrase strand transfer inhibitor; K, thousand; NNRTI, nonnucleoside reverse transcriptase inhibitor; NRTI, nucleoside reverse transcriptase inhibitor; PI, protease inhibitor; VL, viral load.

^a^For a list of antiretroviral abbreviations, full clinical vignette details, as well as specific antiretroviral regimen responses, see Supplementary Tables 1–3.

^b^Second-generation INSTIs were selected by providers (ie, dolutegravir, bictegravir, cabotegravir); no first-generation INSTIs were selected (ie, elvitegravir and raltegravir), with the exception of case S2, where the patient was already taking an elvitegravir-containing regimen; boosted darunavir was selected in all cases as the PI.

^c^Cabotegravir/rilpivirine use as follows: S1, n = 11 (14.3%); S2, n = 12 (16.9%); S3, n = 14 (24.6%); S4, n = 1 (1.7%); S5, n = 30 (52.6%).

Conversely, in case S2 of a patient who was virally suppressed while taking EVG/c/TAF/FTC (lower genetic barrier to resistance) and had a genotypic history of an isolated M184V, the most frequent recommendation (38/71, 53.5%) was to switch to BIC/TAF/FTC (ie, INSTI with higher barrier to resistance), while only 16 (22.5%) chose to maintain the current regimen; in contrast, 12 (16.9%) selected CAB/RPV and none intensified the regimen (Table 3).

Finally, case S3 involved an individual with a suppressed viral load who was taking BIC/TAF/FTC with prior genotypes showing M184V and was experiencing pill and treatment fatigue. Among 57 respondents, there were 5 unique ARV regimens (8.8% heterogeneity), of which maintaining BIC/TAF/FTC was the most common recommendation (n = 40, 70.2%; GSS 2.33; Tables 3 and 4, Supplementary Table 3). Fourteen (24.6%) providers advised switching to CAB/RPV despite a history of treatment failure (GSS, 2).

#### Moderate to Extensive NRTI Resistance (TAMs or K65R) Without INSTI Resistance

##### Viremic Cases

We assessed ARV selection in treatment-experienced persons with detectable viremia, with 2 patterns of moderate to high-level NRTI resistance on cumulative genotypes (ie, TXF) but without known INSTI or PI resistance.

In case V3 (Table 2) of ARV reinitiation following intermittent adherence to EFV/TDF/FTC and with genotypes detecting K65R and M184V (net moderate TXF resistance), there were 21 unique regimens (median GSS, 2.0) among 53 respondents (39.6% heterogeneity) with no single approach representing a majority of providers (Table 3). The most common regimen by class consisted of 1 PI with 1 INSTI and 1 NNRTI (ie, doravirine + DRV/b + DTG, DRV/r + DTG/RPV; 12/53), and the majority (n = 28, 52.8%) chose an NRTI-sparing regimen; in addition, 19 (17%) providers suggested a regimen with >3 drugs (eg, BIC/TAF/FTC + DRV/b). The second-most common regimen by class was 1 INSTI with 2 NRTIs (n = 11, 20.8%) with participants most frequently selecting BIC/TAF/FTC (n = 10, 18.9%; GSS, 1.33) and 1 PI (DRV/b) and 1 INSTI (n = 11, 20.8%).

In a similar scenario (case V4, Table 2) with different NRTI resistance patterns (M184V and TAMs), there were 24 unique ARV regimens from 70 respondents (median GSS, 2.0; 34.3% heterogeneity). A lower proportion chose a strategy of 2 NRTIs + 1 INSTI (n = 10, 14%); 50% (n = 35) suggested an NRTI-sparing regimen (n = 35, 50%), most frequently a 2-drug regimen (n = 28, 40.0%), such as an 1 INSTI + 1 PI (n = 18, DRV/b + DTG) or 1 INSTI + 1 NNRTI (CAB/RPV, n = 6; DTG/RPV, n = 3; Table 3, Supplementary Table 3).

##### Suppressed Cases

We explored ARV selection in 2 case scenarios with moderate to extensive NRTI resistance (without INSTI or PI resistance) but with current suppression. In case S4, a suppressed individual (TDF/FTC + DTG) had an archived genotype showing K65R, M184V, and K103N. Among 54 responses, there were 14 unique provider ARV regimens (26% heterogeneity), of which the majority recommended staying on the current regimen or prescribing a within-class INSTI (GSS, 1.33; n = 28, 51.9%; Table 3). NRTI-sparing regimens were indicated in 20 responses (36%, GSS ≥2.0), including DTG/RPV (n = 15, 27.8%) and CAB/RPV (n = 1, 1.7%).

In case S5—viral suppression with DRV/r + TDF/FTC, a desire for simplification, and a history of TAMs and M184V—there were 8 unique responses among 57 survey participants (14% heterogeneity). A majority (n = 37, 64.9%) suggested simplification to an INSTI/NNRTI regimen (including CAB/RPV; n = 30, 52%), while 7 (12.3%) would continue the current or similar regimen (DRV/b + TXF/FTC) and 5.3% (n = 3) would intensify it (Supplementary Table 3).

#### INSTI Resistance Mutations

##### INSTI Mutation Score <10: Fully Active INSTI

First, we explored ARV decisions in the setting of a minor DTG or BIC mutation where these agents were still classified as “fully active” by the Stanford HDRD (Table 2, case V5; high-level RAL resistance). In this setting, with concomitant moderate to high-level resistance to NRTIs but fully active PI, there were 26 ARV regimens (median GSS, 2.33) among 68 responses (38.2% heterogeneity; Supplementary Table 3). Overall, the majority (n = 60, 88.2%) selected an INSTI-containing regimen, but there was heterogeneity in companion drugs. A total of 31 (45.6%) chose an NRTI-sparing regimen while 6 (8.8%) selected a 2-NRTI + INSTI regimen; 25 (36.8%) suggested a regimen with >3 drugs and 2 (2.9%) recommended use of CAB/RPV.

##### INSTI Mutation Score of 10–14: Potential Low-Level Resistance

In the setting of potential low-level INSTI resistance (failing EVG/c/TAF/FTC; E92Q) with limited NRTI resistance (isolated M184V; Table 2, case V6), there were 19 unique ARV regimens among 77 respondents (24.7% heterogeneity). As compared with cases involving INSTI mutation scores <10, fewer providers (n = 48, 62.3%) included an INSTI in the regimen. The most common approach was to suggest switching a regimen of 2 NRTIs + PI (n = 24, 31.6%) or >3 drugs (n = 25, 32.5%). Only 8 (10.4%) suggested an NRTI-sparing regimen (Supplementary Table 3). Four (5.2%) participants recommended use of CAB/RPV.

We explored potential low-level INSTI resistance in conjunction with more extensive NRTI resistance in 2 cases with PI resistance that was low (Table 4, case V7, n = 62 responses) or moderate (Table 2, case V8, n = 61). In this setting, there was extreme variability in treatment approaches, with 69.5% (43/62) and 75.4% (46/61) heterogeneity, respectively.

##### INSTI Mutation Score of ≥15: Moderate to High Level Resistance

Finally, we explored ARV selection in the setting of moderate to high-level INSTI resistance (cases V9–V11). Across 3 cases, there were 181 responses and 127 unique ARV combinations chosen (70.1% heterogeneity).

## DISCUSSION

This study of HIV provider practices throughout the United States highlights diverse approaches to ARV selection in persons with HIVDR. Unsurprisingly, we found increasing heterogeneity (ie, a larger number of unique ARV regimens selected) and a lack of consensus on favored regimens in settings with more extensive resistance mutations, particularly INSTI resistance. Importantly, several practice patterns could be gleaned from our study, such as the approach to NRTI resistance in suppressed or viremic cases. We also noted consideration of CAB/RPV in patients with treatment experience, suggesting a growing interest in employing this regimen outside of the approved indication. To our knowledge, this is one of the largest surveys of HIV provider practice patterns and helps to characterize approaches when resistance is encountered; it also provides insights into areas where clinical practice guidelines could be enhanced to provide further support to clinicians.

### Approach to an Isolated M184V in Viremic and Suppressed Cases

In the approach to regimen construction in viremic and suppressed cases with an isolated or suspected M184V, most providers appear comfortable using a high-barrier-to-resistance INSTI (including BIC) and 2 NRTIs, with relatively little heterogeneity in specific regimens. In contrast, there was more variability in cases with extensive NRTI resistance, with differences in the approach to patients with ongoing viremia as compared with those who were suppressed. Providers appeared comfortable with regimens containing <2 drugs with predicted activity (ie, GSS <2.0) in the setting of treatment simplification (virally suppressed), despite a history of extensive NRTI resistance. This finding aligns with guidelines from the US Department of Health and Human Services (DHHS): in cases of NRTI resistance, 2 NRTIs plus a fully active high-barrier-to-resistance drug should be included [9, 10]. Most recently, the 2SD study in Kenya showed that among patients whose first-line regimen of 2 NRTIs and an NNRTI failed but who became virally suppressed while taking a ritonavir-boosted PI-based regimen, a subsequent switch to a DTG + 2-NRTI regimen was noninferior [15]. It is presumed from these studies and others that viral suppression is maintained by inclusion of a high-barrier-to-resistance drug, despite resistance to companion drugs. Additionally, there is increasing evidence to suggest that NRTIs maintain antiviral activity, despite significant predicted genotypic resistance (moderate to high level) [16–18].

### Approach to Moderate to High-Level NRTI Resistance in Viremic and Suppressed Cases

Conversely, most respondents suggested an NRTI-sparing regimen or a regimen with >3 drugs when presented with patients who had treatment failure with first-line NNRTI, with accompanying extensive NRTI resistance and viremia. Providers elected to initiate an NRTI-sparing regimen (mean, 51%), with only 17.6% of providers (mean across vignettes) selecting a 2-NRTI + INSTI–containing regimen (GSS, 1.33). This likely represents less certainty to the number of genotypically active companion drugs required in a regimen with a fully active INSTI or PI in viremic cases. Consequently, case scenarios with treatment failure and a moderate to high level of NRTI resistance generated a high degree of heterogeneity (35%–40%). Guidelines (DHHS and International Antiviral Society–USA) recommend that in a history of failure with NNRTI + NRTI regimens, providers use a regimen containing fully active boosted PI or DTG + 2 NRTIs, 1 of which is fully active [9, 10]. In the DAWNING trial, patients with virologic failure while taking an NNRTI-based regimen received either lopinavir/ritonavir or DTG paired with 2 NRTIs, 1 of which was fully active. DTG was superior to lopinavir/ritonavir, although resistance did develop for 2 of 11 patients in the DTG arm [5]. The NADIA trial has recently suggested that <2 predicted fully active drugs may be needed after virologic failure on first-line NNRTI plus lamivudine or emtricitabine with TXF disoproxil fumarate [6]. In our 2 survey cases with extensive NRTI resistance (including TXF) and viremia, the predicted median GSS of selected regimens was 2.0, and a mean 35.5% of providers selected a 2-drug regimen (Table 3). This practice pattern suggests that in cases of first-line NNRTI resistance with extensive NRTI resistance, providers select an approach of using a regimen that has 2 fully active drugs (ie, other than NRTIs) with 1 high-barrier-to-resistance drug.

### Approach to Treatment Failure and CAB/RPV Use in Suppressed and Viremia Cases

While long-acting ARV (ie, CAB/RPV) is approved in individuals who are virally suppressed without known resistance, there is increasing interest in its use in nonadherent or hard-to-reach populations, who are often viremic at the time of treatment initiation with a history of resistance [19]. The main published experience supporting its use in these cases comes from Christopoulos et al, who initiated CAB/RPV in 15 viremic cases, with 12 achieving viral suppression, including 1 with baseline INSTI mutation (N155H, low-level resistance) [20], with additional reports of compassionate use [21]. Guidelines do identify CAB/RPV as a treatment simplification strategy but say, “Criteria for use should include individuals who have good adherence and engagement in care, with no baseline resistance to either medication, no prior virologic failure” [10]. In our study, in 3 cases of treatment simplification with a history of virologic failure (cases S1–S3), providers expressed interest in using this regimen, with CAB/RPV in the top 3 selected regimens. Furthermore, while CAB/RPV is not recommended in patients with viremia and treatment failure and did not reflect a majority preference for any vignette in this study, we found that CAB/RPV was a selected regimen by a few providers in scenarios of treatment failure and ongoing viremia (cases V4–V6). This suggests a growing interest in use of CAB/RPV outside of current narrow indications and recommendations.

### Approach to INSTI Resistance

An area of ongoing uncertainty and limited guidance is the approach to INSTI resistance mutations. In settings with “susceptibility” to DTG, DHHS guidelines suggest using a boosted PI with 2 NRTIs, DTG twice daily with 2 NRTIs, or DTG twice daily with a boosted PI [10]. However, defining susceptibility poses challenges, as exposure to raltegravir or elvitegravir can select for INSTI mutations in which BIC or DTG is still regarded as fully “active” or “potential low-level resistance” on phenotypic and genotypic reports. We sought to understand provider comfort with maintaining an INSTI in treatment regimens in such situations. We found substantial heterogeneity when approaching INSTI resistance mutations. With mutations conferring Stanford HDRD scores <10 (ie, T97a), the majority chose regimens inclusive of an INSTI (88.2%), but many (36.8%) chose to intensify to >3 drugs. Providers considered alternative anchor drugs with progressive INSTI resistance: when presented vignettes with “potential low-level INSTI resistance” (Stanford HDRD score, 10–14) with limited NRTI resistance, a majority (62.3%) included INSTIs in the regimen and a similar proportion chose to intensify to >3 drugs. We found less consistency when such low-level resistance INSTI mutations were considered with concomitant NRTI resistance; across case scenarios, there was 69.5%–75.4% heterogeneity (ie, a large proportion of unique regimens suggested) with such multiclass resistance.

### Limitations

Our study had limitations. Lenacapavir was not yet approved by the Food and Drug Administration during the study period and was not indicated as a treatment option, although a few respondents did cite this drug as an “other” option. Additionally, our study is subject to possible hypothetical bias, where there could be a difference in how providers respond to hypothetical cases when compared with real-life practice. Use of the GSS as a measure of a regimen's predicted activity does not account for the fact that some drugs in a regimen are more potent than others (ie, PIs and INSTIs); therefore, this is a rough assessment of a regimen's activity. Our study respondents were predominantly infectious disease physicians at academic settings and may not reflect the growing proportion of HIV care delivered by providers without HIV training [22–25]. We were unable to assess differences in practice patterns by US region, given the limited number of survey responses from several regions. Finally, our survey did not allow for a shared decision-making strategy that providers often employ, whereby they select a few ARV regimens and incorporate the patient's perspective when crafting a final regimen.

## Conclusions

In this national provider survey, through systematic use of a series of clinical case vignettes, we sought to clarify an approach to ARV resistance mutations frequently seen in practice. In cases with low heterogeneity, this study presents a consensus to management of HIV resistance with potential alternative options. We were able to shed light on provider practice patterns, and the responses to survey questions could be used as an educational tool for ARV decision making.

## Supplementary Material

ofad541_Supplementary_Data

## Supplementary Data

Supplementary materials are available at *Open Forum Infectious Diseases* online. Consisting of data provided by the authors to benefit the reader, the posted materials are not copyedited and are the sole responsibility of the authors, so questions or comments should be addressed to the corresponding author.

## Notes

***Acknowledgments.*** The authors would like to thank the participants.

***Author contributions.*** C. K. L., M. S., and M. B. M. R. contributed to study design and development. M. S., M. B. M. R., S. B., and S. K. conducted data collection and analysis. All authors contributed to data interpretation and manuscript writing.

***Financial support.*** The study was supported by an internal Johns Hopkins faculty gift account (to M. S.) and an educational grant from HIV-ASSIST Inc (a registered 501c3 nonprofit corporation) to Johns Hopkins for supporting HIV educational activities.
