# Supplementary material for: A National HIV Provider Survey of Antiretroviral Therapy Preferences for Management of Treatment-Naive and Experienced Individuals With Drug Resistance
Source: Open Forum Infect Dis. 2023 Oct 31;10(11):ofad541. doi: 10.1093/ofid/ofad541 (PMC10655941; doi:10.1093/ofid/ofad541)
Supplement: ofad541_Supplementary_Data [file ofad541_supplementary_data.docx]

Supplemental Methods

Supplemental Table 1. Full list of antiretroviral abbreviations.

| Name | Abbreviation | Name | Abbreviation |
| --- | --- | --- | --- |
| Abacavir | ABC | Elvitegravir/cobicistat | EVG/c |
| Atazanavir-ritonavir | ATV/r | Emtricitabine | FTC |
| Bictegravir | BIC | Etravirine | ETR |
| Cabotegravir | CAB | Fostemsavir | FOS |
| Darunavir | DRV | Ibalizumab | IBA |
| Darunavir/cobicistat | DRV/c | Lamivudine | 3TC |
| Darunavir/ritonavir | DRV/r | Lencapavir | LEN |
| Darunavir/ritonavir twice daily | DRV/r BID | Maraviroc | MVC |
| Dolutegravir | DTG | Raltegravir | RAL |
| Dolutegravir twice daily | DTG BID | Rilpivirine | RPV |
| Efavirenz | EFV | Tenofovir alafenamide | TAF |
| Elvitegravir | EVG | Tenofovir disoproxil | TDF |

Supplemental Table 2. Full clinical vignette by case.

| Case | Vignette |
| --- | --- |
| V1 | A 23-year-old female, who was intermittently taking Truvada (TDF/FTC) for PrEP, is newly diagnosed with HIV. Her baseline genotype testing shows an M184V mutation. Her CD4 is >200 and VL is 125,000.    HIV viral load: 125,000 copies/mL CD4 count: >200 cells/mm3 Creatinine clearance (CrCl): >60 mL/min Co-morbidities: none Co-medications: none HLA-B5701: negative Prior genotype: N/A Current genotype: NRTI- M184V Treatment history: Truvada (TDF/FTC) Current Regimen: none Tropism: unknown |
| V2 | A 53-year-old male presents with a new diagnosis of HIV, and is being considered for rapid ART initiation. His CD4 is 150 and his viral load is 400,000. A baseline genotype test is pending. He has a history of taking HIV pre-exposure prophylaxis (PrEP), but has not taken it consistently for several months. The patient would like to be initiated on ART the same day.    HIV viral load: 400,000 copies/mL CD4 count: 150 cells/mm3 Creatine clearance (CrCl): >60 mL/min Co-morbidities: none Co-medications: none HLA-B5701: negative Prior genotype: N/A Current genotype: pending Treatment history: Truvada (TDF/FTC) Current Regimen: none Tropism: unknown |
| V3 | A 36-year-old male recently immigrated to the US from South Africa.  He had been taking EFV/TDF/FTC for several years, but recently had intermittent adherence while trying to establish care in his new city.  A baseline viral load is obtained and is 170,000 and CD4 count is 210.  A baseline genotype is listed below.  HIV viral load: 170,000 copies/mL CD4 count: 210 cells/mm3 Creatinine clearance (CrCl): >60 mL/min  Co-morbidities: none Co-medications: none HLA-B5701: negative Prior genotype: unknown Current genotype: NRTI - M184V, K65R, Y115F NNRTI - K103N Treatment history: Atripla (EFV/TDF/FTC) Current Regimen: none Tropism: unknown |
| V4 | A 34-year-old male presents after being lost to follow up and is off medications. He was diagnosed with HIV more than 12 years ago, previously treated with Lopinavir/ritonavir (LPV/r) and Combivir (3TC/AZT), as well as with Atripla (EFV/TDF/FTC). He is known to have an M184V mutation and thymidine-associated mutations (TAMs). He now has a CD4 count less than 100 and a viral load of 110,000. His available genotypes are listed below.  He would prefer as few pills as possible, and is open to long acting and injectable medications.  HIV viral load: 110,000 copies/mL CD4 count: 95 cells/mm3 Creatinine clearance (CrCl): >60 mL/min Co-morbidities: none Co-medications: none HLA-B5701: negative Prior genotype: M184V, M41L, T215Y, L210W Current genotype:  NRTI - M184V, M41L, T215Y, L210W Treatment history: LPV/r + Combivir (3TC/AZT), Atripla (EFV/TDF/FTC) Current Regimen: none Tropism: unknown |
| V5 | A 37-year-old male with HIV presents to your clinic after many years of intermittent care in another state.  He is unable to provide details of his prior treatment.  A baseline genotype reveals several mutations that suggest NRTI, PI, INSTI and NNRTI exposure. He has a CD4 count of 184 and a viral load of 55,000.  He prefers as few pills as possible, and is willing to consider any treatment options including usage of injectable medications.  HIV viral load: 55,000 copies/mL CD4 count: 184 cells/mm3 Creatinine clearance (CrCl): >60 mL/min Co-morbidities: none Co-medications: none HLA-B5701: negative Prior genotype: unknown Current genotype:  NRTI - M184V, M41L, D67N, L74V, L210W, T215D, K219N  NNRTI - K103N  PI - L90M  INSTI- Y143C, T97A  Treatment history: unknown Current Regimen: none Tropism: unknown |
| V6 | A 48-year-old male with HIV is referred to your clinic by his primary care provider, after annual labs showed HIV viremia while on Stribild (EVG/cTAF/FTC). He has a history of intermittent adherence and an M184V mutation. His viral load is currently 310,000 and his CD4 count is less than 200. A genotype is done and is notable for an E92Q mutation. He is willing to consider any treatment options, including usage of injectable medications.  HIV viral load: 310,000 copies/mL CD4 count: <200 cells/mm3 Creatinine clearance (CrCl): >60 mL/min Co-morbidities: none Co-medications: none HLA-B5701: negative Prior genotype:  NRTI - M184V Current genotype:  NRTI - M184V  INSTI - E92Q Treatment history: Stribild (EVG/c/TAF/FTC) Current Regimen: none Tropism: unknown |
| V7 | A 60-year-old man was initially diagnosed with HIV in 1992. He had multiple ART regimens and had clinical improvement but never achieved viral suppression until 2007.  He had extensive NRTI, NNRTI, PI resistance (see below) and was initiated on Darunavir/ritonavir (DRV/r) twice daily, Raltegravir (RAL) twice daily, and Truvada (TDF/FTC). He achieved viral suppression for several years. On annual follow-up he is now found to have viremia, and he mentions that he occasionally forgets to take evening doses of his regimen. His viral load is 12,000 and CD4 count has declined. A repeat genotype reveals additional resistance (see below). He is willing to stay on the same regimen or change based on your suggestions, and is open to injectable medications.   HIV viral load: 12,000 copies/mL CD4 count: 196 cells/mm3 Creatinine clearance (CrCl): >60 mL/min Co-morbidities: none Co-medications: none HLA-B5701: negative Prior genotype: NRTI- M41L, D67N, T69A, K70R, L74V, M184V, T215Y, K219Q NNRTI- K101P, K103N PI- M46I, I54V, V82T, L90M Current genotype:  NRTI - M41L, D67N, T69A, K70R, L74V, M184V, T215Y, K219Q  NNRTI- K101P, K103N  PI-I54V, I84V (new)  INSTI - E92Q (new) Treatment history: Multiple regimens, but specific details are unavailable Current Regimen: Darunavir/ritonavir BID (DRV/r BID) + Raltegravir BID (RAL BID) + Truvada (TDF/FTC) Tropism: unknown |
| V8 | A 44-year-old male with HIV presented to care after hospitalization for *Pneumocystis* pneumonia. He had been intermittently adherent to ART in the past. During hospitalization, he was found to have a CD4 count of 65 and a viral load of 200,000. Genotype is performed and reveals several mutations in multiple drug classes; a summary of all available prior genotypes is listed below. In an effort to improve adherence, inpatient providers initiated once daily Biktarvy (BIC/TAF/FTC). The patient is seen in follow-up and reports good adherence. Six months after ART initiation his viral load remains detectable at 185,000 copies/ml. A repeat genotype reveals additional NRTI mutations listed below. Given the resistance profile, which treatment regimen would you prescribe? The patient is amenable to injectable therapy.   HIV viral load: 185,000 copies/mL CD4 count: 85 cells/mm3 Creatinine clearance (CrCl): >60 mL/min Co-morbidities: none Co-medications: none HLA-B5701: negative Prior genotypes:  NRTI - D67N, K70R, M184V NNRTI - K101E, V108I PI - L33F, I50V, I54L INSTI - T97A, N155H Current genotype: NRTI - D67N, K70R, M184V, K65R (new) NNRTI - K101E, V108I  PI - L33F, I50V, I54L INSTI - T97A, N155H  Treatment history: unknown Current Regimen: none Tropism: Dual-mixed |
| V9 | A 46-year-old female with HIV (diagnosed >10 years ago) on ART, and a past medical history of coronary artery disease (CAD) presents for routine follow-up. Her labs reveal her CD4 count has dropped to less than 50, and her HIV viral load is 430,000 copies/ml.  Available records show she has previously experienced viremia while prescribed Atripla, as well as Truvada (TDF/FTC) and Raltegravir.  Full treatment details of other regimens, and genotypes were not available, but available data is summarized below. She is currently taking Etravirine, Dolutegravir, Darunavir-ritonavir along with tenofovir/emtricitabine and reports good adherence.  She is willing to consider any treatment options, including usage of injectable medications.  HIV viral load: 430,000 copies/mL CD4 count: <50 cells/mm3 Creatinine clearance (CrCl): >60 mL/min Co-morbidities: CAD Co-medications: none HLA-B5701: negative Available prior genotype: M184V, K103N, T215F, M41L, I84V, Q148H Current genotype: NRTI - M41L, D67N, T69D, K70Q, M184V, T215F, L74I NNRTI - Y181C, K103N PI - V32I, L33F, M46L, I54V, I84V, L90M INSTI - G140S, Q148H Other mutations - K20R, T39A, K219H ​​ Known treatment history: Atripla (EFV/TDF/FTC),  Raltegravir (RAL) + Tenofovir (TDF)/Emtricitabine (FTC)  Current Regimen: Etravirine (ETR) + Dolutegravir (DTG) + Darunavir-ritonavir (DRV/r) + Tenofovir (TAF)/Emtricitabine (FTC) Tropism: dual tropic |
| V10 | 28-year-old female presents with perinatally acquired HIV, treated with numerous different regimens.  At approximately age 18, she was started on a regimen of Raltegravir with Etravirine and Truvada (TDF/FTC) on which she was intermittently suppressed, with periods of stopping medications. She has been on several regimens in the interim but has had long periods out of care. Most recently, she was placed on Dolutegravir (DTG) + Odefsey (RPV/TAF/FTC), with a stated preference for smaller and fewer pills. She previously tried PIs several times (recently DRV/c) with poor tolerance due to nausea/vomiting and difficulty swallowing pills, but she is willing to consider them again. She is now presenting with an increased viral load (ranging 10,000-15,000) and a decreased CD4 count to 180.  She is open to staying on her current regimen, or switching based on your recommendations and is open to considering injectable medications.   HIV viral load: 10,000-15,000 copies/mL CD4 count: 180 cells/mm3 Creatinine clearance (CrCl): >60 mL/min  Co-morbidities: none Co-medications: none HLA-B5701: unknown Cumulative available genotypes: NRTI - M41L, M184V, T215Y NNRTI - K101P, V106I, V179F, Y181C PI - V82A, L90M, K20T INSTI - E92Q, G140S, Q148R Other mutations - T69I  Treatment history: List of drugs to which she has been exposed:  NNRTI-Etravirine (ETR), Rilpivirine (RPV)  INSTI- Raltegravir (RAL), Dolutegravir (DTG), Elvitegravir (EVG)  NRTI-Tenofovir (TAF/TDF), Emtricitabine (FTC), Zidovudine (AZT), Zidovudine/Lamivudine (AZT/3TC), Abacavir/Lamivudine (ABC/3TC)  PI--Darunavir-cobicistat (DRV/c), Darunavir-ritonavir (DRV/r), Atazanavir (ATV), Lopinavir-ritonavir (LPV/r) Current Regimen: Dolutegravir (DTG) + Odefsey (RPV/TAF/FTC) Tropism: X4 |
| V11 | 49-year-old male with HIV presents with a long and complex treatment history. He has a history of social issues affecting his care (including experiencing homelessness, lack of familial support, methamphetamine use) and loss to follow up. His CD4 count is 38 and VL is 32,500. He has most recently been on darunavir/ritonavir (DRV/r) twice daily, dolutegravir (DTG) BID, and Truvada (TDF/FTC), but has continued to have a detectable viral load. Mutations from all genotypes are shown below, along with ARVs that the patient has previously taken.  HIV viral load: 32,500 copies/mL CD4 count: 38 cells/mm3 Creatinine clearance (CrCl): >60 mL/min  Co-morbidities: none Co-medications: none HLA-B5701: negative Cumulative genotypes: NRTI - M41L, E44A, T74P, V75M, F77L, M184V, L210W, T215Y NNRTI - K103N, G190A PI - V32I, L33F, I54L, I84V  INSTI - G140S, Q148H  Other mutations - V11I, V118V/I Treatment history: Prior treatment exposure to the following drugs- NRTIs: Zidovudine (AZT), Lamivudine (3TC), stavudine (D4T), Abacavir (ABC) NNRTI: Nevirapine (NVP), Efavirenz(EFV), Etravirine (ETR),  PI (boosted): Indinavir (IDV), Amprenavir (APV), Lopinavir (LPV), Tipranavir (TPV), Darunavir (DRV) INSTI: Raltegravir (RAL), Dolutegravir (DTG) Current Regimen: Darunavir-ritonavir (DRV/r) BID + Dolutegravir (DTG) BID + Truvada (TDF/FTC) daily Tropism: R5 |
| S1 | A 43-year-old male transfers to your clinic after a recent move, with a history of HIV and hyperlipidemia. He reports being on prior ART regimens, but cannot recall the specific medications, and reports periods during which he was out of care in the past. Two years ago, he re-engaged with HIV care with another provider and a baseline genotype showed multiple mutations: K103N, M184. Prior genotypes were unavailable. The previous provider had started him on DRV/c/TAF/FTC+DTG. He has been virally suppressed consistently on this current regimen with a CD4 >200, and good adherence. His preference is for smaller and/or fewer pills, if possible. The patient is open to continuing the current regimen or changing the regimen based on your recommendation, and is open to injectable medications.   HIV viral load: <20 copies/mL CD4 count: >200 cells/mm3 Creatinine clearance (CrCl): >60 mL/min  Co-morbidities: Hyperlipidemia Co-medications: Atorvastatin HLA-B5701: negative Prior genotype: unknown Most recent genotype: NRTI - M184V NNRTI - K103N Treatment history: unknown Current regimen: Tivicay (DTG) + Symtuza (DRV/c/TAF/FTC) Tropism: unknown |
| S2 | A 37-year-old male diagnosed with HIV 15 years ago presents to your care with no known co-morbid conditions. He has a history of treatment failure on Atripla (EFV/TDF/FTC), but he has been suppressed on Genvoya (EVG/c/TAF/FTC) for several years. His CD4 is 375. Upon reviewing records you note a prior genotype while on EFV/TDF/FTC which showed an isolated M184V.  The patient is open to continuing the current regimen or changing the regimen based on your recommendation, and is open to injectable medications.  HIV viral load: <20 copies/mL CD4 count: 375 cells/mm3 Creatinine clearance (CrCl): >60 mL/min  Co-morbidities: none Co-medications: none HLA-B5701: negative Prior genotype: NRTI - M184V  Current genotype: none  Treatment history: Atripla (EFV/TDF/FTC) Current Regimen: Genvoya (EVG/c/TAF/FTC) Tropism: unknown |
| S3 | A 24-year-old female presents with perinatally acquired HIV. She is new to your office and requests a regimen with as few pills as possible. She acknowledges a prior history of non-adherence and reports being on multiple different regimens when she was younger.  More recently, she has been consistently engaged in care at another clinic for several years and has good social support. She has been virally suppressed on her current regimen of BIC/TAF/FTC for two years; a genotype prior to starting this regimen showed an M184V, but no other mutations. The patient prefers as few pills as possible, is open to continuing the current regimen or changing the regimen based on your recommendation, and is open to injectable medications.   HIV viral load: <20 copies/mL CD4 Count: >200 cells/mm3 Creatinine clearance (CrCl): >60 mL/min  Co-morbidities: none Co-medications: none HLA-B5701: unknown Prior genotypes: Unavailable Most recent genotype prior to current regimen: NRTI - M184V Available Treatment history: Zidovudine (AZT); Trizivir (AZT/ABC/3TC); Lopinavir-ritonavir (LPV/r) + Combivir (AZT/3TC); Atripla (EFV/TDF/FTC); Atanzavir-ritonavir (ATV/r) + Truvada (TDF/FTC) Current Regimen: Biktarvy (TAF/FTC/BIC) Tropism: unknown |
| S4 | A 64-year-old female, originally from Zimbabwe, has been virally suppressed on DTG+TDF/FTC for 2 years.  She indicates she was previously on EFV/TDF/FTC, but reports she was switched several years ago by her providers but she is unaware of the reasons.  She is post-menopausal and has hyperlipidemia (HLD), and major depressive disorder (MDD). Her CD4 count is 380 and her viral load is undetectable. No prior genotypes are available. An HIV proviral DNA genotype (i.e., archive DNA genotype) shows multiple mutations: K65R, K103N, M184V. The patient is open to continuing the current regimen or changing the regimen based on your recommendation.  HIV viral load: <20 copies/mL CD4 count: 380 cells/mm3 Creatinine clearance (CrCl): >60 mL/min  Co-morbidities:  HLD, MDD Co-medications: Sertraline, Atorvastatin HLA-B5701: negative Prior genotype: none HIV DNA/Archived genotype: K65R, K103N, M184V Treatment history: Atripla (EFV/TDF/FTC) Current Regimen: Dolutegravir (DTG) + Tenofovir/emtricitabine (TDF/FTC) Tropism: unknown |
| S5 | A 36-year-old female presents with a 10-year history of HIV, initially diagnosed after having pneumocystis pneumonia with a low CD4+ cell count and a high viral load. She was started on Atripla (EFV/TDF/FTC) with intermittent adherence, and was lost to follow up. She returned to care 4 years ago, and was noted to have multiple resistance mutations on genotypic analysis: M41L, T215Y, L210W, M184V.  She was started on Darunavir/ritonavir and Truvada (TDF/FTC) and has had  an undetectable viral load for several years, and a CD4 cell count of 440. She has seen advertisements for one-pill daily treatments, and has heard of longer acting injectable medications and inquires about these options. She notes some diarrhea and GI side-effects that she attributes to her current oral regimen. The patient is open to continuing the current regimen or changing the regimen based on your recommendation. Her preference is for as few pills as possible, and is open to long-acting injectable medications if you think it is appropriate.  HIV viral load: <20 copies/mL CD4 count: 440 cells/mm3 Creatinine clearance (CrCl): >60 mL/min  Co-morbidities: GI side effects Co-medications: none HLA-B5701: negative Prior genotypes: none Genotype prior to starting current regimen: NRTI - M184V,M41L,T215Y, L210W Treatment history: Atripla (EFV/TDF/FTC) Current Regimen: Darunavir/ritonavir (DRV/r), Tenofovir/emtricitabine (TDF/FTC) Tropism: unknown |

Supplemental Table 3. Full responses to case-vignettes with resistance.

| Case | Summary of Response by Regimen | Summary of Response by Class n (%) |
| --- | --- | --- |
| V1 | \|  \| Freq. \| Percent \| \| --- \| --- \| --- \| \| BIC/TAF/FTC \| 55 \| 77.46 \| \| DTG,DRV/c/TAF/FTC \| 3 \| 4.23 \| \| DTG,TAF/FTC \| 3 \| 4.23 \| \| BIC/TAF/FTC,DRV/c \| 2 \| 2.82 \| \| DTG/ABC/3TC \| 2 \| 2.82 \| \| DRV/c/TAF/FTC \| 1 \| 1.41 \| \| DRV/r,BIC/TAF/FTC \| 1 \| 1.41 \| \| DTG,DRV/r,TAF/FTC \| 1 \| 1.41 \| \| DTG,TDF/FTC \| 1 \| 1.41 \| \| DTG/RPV \| 1 \| 1.41 \| \| Other \| 1 \| 1.41 \| \| Total \| 71 \| 100.00 \| | \|  \| Freq. \| Percent \| \| --- \| --- \| --- \| \| 1 INSTI,2 NRTI \| 61 \| 87.14 \| \| 1 PI,1 INSTI,2 NRTI \| 7 \| 10.00 \| \| 1 INSTI,1 NNRTI \| 1 \| 1.43 \| \| 1 PI,2 NRTI \| 1 \| 1.43 \| \| Total \| 70 \| 100.00 \| |
| V2 | \|  \|  \|  \|  \| Freq. \| Percent \| \| --- \| --- \| --- \| --- \| --- \| --- \| \|  \|  \|  \| BIC/TAF/FTC \| 45 \| 62.50 \| \|  \|  \|  \| DRV/c/TAF/FTC \| 9 \| 12.50 \| \|  \|  \|  \| DTG,DRV/c/TAF/FTC \| 6 \| 8.33 \| \|  \|  \|  \| BIC/TAF/FTC,DRV/c \| 3 \| 4.17 \| \|  \|  \|  \| DTG,TAF/FTC \| 3 \| 4.17 \| \|  \|  \|  \| DTG/ABC/3TC \| 2 \| 2.78 \| \|  \|  \|  \| DTG BID,DRV/c/TAF/FTC \| 1 \| 1.39 \| \|  \|  \|  \| DTG,DRV/c \| 1 \| 1.39 \| \|  \|  \|  \| DTG,TAF/FTC,DRV/c \| 1 \| 1.39 \| \|  \|  \|  \| DTG,TDF/FTC \| 1 \| 1.39 \| \|  \|  \|  \| Total \| 72 \| 100.00 \| | \|  \| Freq. \| Percent \| \| --- \| --- \| --- \| \| 1 INSTI,2 NRTI \| 51 \| 70.83 \| \| 1 PI,1 INSTI,2 NRTI \| 11 \| 15.28 \| \| 1 PI,2 NRTI \| 9 \| 12.50 \| \| 1 PI,1 INSTI \| 1 \| 1.39 \| \| Total \| 72 \| 100.00 \| |
| V3 | \|  \| Freq. \| Percent \| \| --- \| --- \| --- \| \| BIC/TAF/FTC \| 10 \| 18.87 \| \| DTG,DRV/c \| 8 \| 15.09 \| \| DOR,DTG,DRV/c \| 6 \| 11.32 \| \| DTG,DRV/c/TAF/FTC \| 4 \| 7.55 \| \| DTG/RPV,DRV/c \| 3 \| 5.66 \| \| DOR,BIC/TAF/FTC \| 2 \| 3.77 \| \| DOR,DTG \| 2 \| 3.77 \| \| DRV/r,DTG/RPV \| 2 \| 3.77 \| \| DTG BID,DRV/c \| 2 \| 3.77 \| \| DTG/3TC,DRV/c \| 2 \| 3.77 \| \| DTG/RPV \| 2 \| 3.77 \| \| BIC/TAF/FTC,DRV/c \| 1 \| 1.89 \| \| CAB/RPV \| 1 \| 1.89 \| \| DOR,DTG,DRV/r \| 1 \| 1.89 \| \| DRV/c/TAF/FTC \| 1 \| 1.89 \| \| DRV/r,BIC/TAF/FTC \| 1 \| 1.89 \| \| DTG,DRV/r \| 1 \| 1.89 \| \| DTG,DTG/3TC \| 1 \| 1.89 \| \| DTG,RPV/TAF/FTC \| 1 \| 1.89 \| \| DTG/ABC/3TC \| 1 \| 1.89 \| \| FTC,DTG/RPV \| 1 \| 1.89 \| \| Total \| 53 \| 100.00 \| | \|  \| Freq. \| Percent \| \| --- \| --- \| --- \| \| 1 PI,1 INSTI,1 NNRTI \| 12 \| 22.64 \| \| 1 INSTI,2 NRTI \| 11 \| 20.75 \| \| 1 PI,1 INSTI \| 11 \| 20.75 \| \| 1 PI,1 INSTI,2 NRTI \| 6 \| 11.32 \| \| 1 INSTI,1 NNRTI \| 5 \| 9.43 \| \| 1 INSTI,2 NRTI,1 NNRTI \| 3 \| 5.66 \| \| 1 PI,1 INSTI,1 NRTI \| 2 \| 3.77 \| \| 1 INSTI,1 NRTI \| 1 \| 1.89 \| \| 1 INSTI,1 NRTI,1 NNRTI \| 1 \| 1.89 \| \| 1 PI,2 NRTI \| 1 \| 1.89 \| \| Total \| 53 \| 100.00 \| |
| V4 | \|  \| Freq. \| Percent \| \| --- \| --- \| --- \| \| DTG,DRV/c \| 16 \| 22.86 \| \| BIC/TAF/FTC \| 10 \| 14.29 \| \| DTG,DRV/c/TAF/FTC \| 7 \| 10.00 \| \| CAB/RPV \| 6 \| 8.57 \| \| BIC/TAF/FTC,DRV/c \| 3 \| 4.29 \| \| DTG,DOR/TDF/3TC \| 3 \| 4.29 \| \| DTG/RPV \| 3 \| 4.29 \| \| I am unsure or unable to answer \| 3 \| 4.29 \| \| DOR,BIC/TAF/FTC \| 2 \| 2.86 \| \| DOR,DTG,DRV/c \| 2 \| 2.86 \| \| DTG,DRV/r \| 2 \| 2.86 \| \| DOR,DRV/c/TAF/FTC \| 1 \| 1.43 \| \| DOR,DTG,DRV/r \| 1 \| 1.43 \| \| DOR,DTG/3TC \| 1 \| 1.43 \| \| DOR,DTG/ABC/3TC \| 1 \| 1.43 \| \| DRV/c,DTG/RPV \| 1 \| 1.43 \| \| DRV/c/TAF/FTC \| 1 \| 1.43 \| \| DRV/r,DOR/TDF/3TC,BIC/TAF/FTC \| 1 \| 1.43 \| \| DTG/3TC,DRV/c \| 1 \| 1.43 \| \| DTG/RPV,DRV/c \| 1 \| 1.43 \| \| FOS,DRV/c/TAF/FTC \| 1 \| 1.43 \| \| RPV/TAF/FTC \| 1 \| 1.43 \| \| TAF/FTC \| 1 \| 1.43 \| \| TAF/FTC,DTG/RPV \| 1 \| 1.43 \| \| Total \| 70 \| 100.00 \| | \|  \| Freq. \| Percent \| \| --- \| --- \| --- \| \| 1 PI,1 INSTI \| 18 \| 26.87 \| \| 1 INSTI,2 NRTI \| 10 \| 14.93 \| \| 1 PI,1 INSTI,2 NRTI \| 10 \| 14.93 \| \| 1 INSTI,1 NNRTI, \| 9 \| 13.43 \| \| 1 INSTI,2 NRTI,1 NNRTI \| 7 \| 10.45 \| \| 1 PI,1 INSTI,1 NNRTI, \| 6 \| 8.96 \| \| 1 INSTI,1 NRTI,1 NNRTI \| 1 \| 1.49 \| \| 1 PI,1 EI,2 NRTI \| 1 \| 1.49 \| \| 1 PI,1 INSTI,1 NRTI \| 1 \| 1.49 \| \| 1 PI,2 NRTI \| 1 \| 1.49 \| \| 1 PI,2 NRTI,1 NNRTI \| 1 \| 1.49 \| \| 2 NRTI \| 1 \| 1.49 \| \| 2 NRTI,1 NNRTI \| 1 \| 1.49 \| \| Total \| 67 \| 100.00 \| |
| V5 | \|  \| Freq. \| Percent \| \| --- \| --- \| --- \| \| BIC/TAF/FTC,DRV/c \| 7 \| 10.29 \| \| BIC/TAF/FTC \| 6 \| 8.82 \| \| DTG,DRV/c \| 6 \| 8.82 \| \| DTG,DRV/c/TAF/FTC \| 6 \| 8.82 \| \| DTG/RPV,DRV/c \| 5 \| 7.35 \| \| DTG/RPV \| 4 \| 5.88 \| \| DOR,DTG,DRV/c \| 3 \| 4.41 \| \| DRV/c/TAF/FTC \| 3 \| 4.41 \| \| DTG BID,DRV/c \| 3 \| 4.41 \| \| DTG BID,DRV/c/TAF/FTC \| 3 \| 4.41 \| \| CAB/RPV \| 2 \| 2.94 \| \| DOR,BIC/TAF/FTC \| 2 \| 2.94 \| \| DOR,DTG BID,DRV/c \| 2 \| 2.94 \| \| DTG BID,DOR/TDF/3TC \| 2 \| 2.94 \| \| DTG,DRV/r \| 2 \| 2.94 \| \| I am unsure or unable to answer \| 2 \| 2.94 \| \| ABC,BIC/TAF/FTC \| 1 \| 1.47 \| \| DOR,DRV/c/TAF/FTC \| 1 \| 1.47 \| \| DOR,DTG BID,DRV/c/TAF/FTC \| 1 \| 1.47 \| \| DOR,DTG BID,DRV/r \| 1 \| 1.47 \| \| DOR/TDF/3TC \| 1 \| 1.47 \| \| DRV/c,DTG/RPV \| 1 \| 1.47 \| \| DTG BID,DRV/r,TAF/FTC \| 1 \| 1.47 \| \| DTG BID,FOS,DRV/c \| 1 \| 1.47 \| \| DTG,DRV/r,DRV/c \| 1 \| 1.47 \| \| RPV,BIC/TAF/FTC \| 1 \| 1.47 \| \| Total \| 68 \| 100.00 \| | \|  \| Freq. \| Percent \| \| --- \| --- \| --- \| \| 1 PI,1 INSTI,2 NRTI \| 17 \| 25.76 \| \| 1 PI,1 INSTI \| 12 \| 18.18 \| \| 1 PI,1 INSTI,1 NNRTI \| 12 \| 18.18 \| \| 1 INSTI,1 NNRTI \| 6 \| 9.09 \| \| 1 INSTI,2 NRTI \| 6 \| 9.09 \| \| 1 INSTI,2 NRTI,1 NNRTI \| 5 \| 7.58 \| \| 1 PI,2 NRTI \| 3 \| 4.55 \| \| 1 INSTI,3 NRTI \| 1 \| 1.52 \| \| 1 PI,1 INSTI,1 EI \| 1 \| 1.52 \| \| 1 PI,1 INSTI,2 NRTI,1 NNRTI \| 1 \| 1.52 \| \| 1 PI,2 NRTI,1 NNRTI \| 1 \| 1.52 \| \| 2 NRTI,1 NNRTI \| 1 \| 1.52 \| \| Total \| 66 \| 100.00 \| |
| V6 | \|  \| Freq. \| Percent \| \| --- \| --- \| --- \| \| DRV/c/TAF/FTC \| 23 \| 29.87 \| \| BIC/TAF/FTC \| 14 \| 18.18 \| \| DTG BID,DRV/c/TAF/FTC \| 8 \| 10.39 \| \| BIC/TAF/FTC,DRV/c \| 7 \| 9.09 \| \| DTG,DRV/c/TAF/FTC \| 5 \| 6.49 \| \| CAB/RPV \| 4 \| 5.19 \| \| DTG BID,TAF/FTC \| 3 \| 3.90 \| \| DOR,DRV/c/TAF/FTC \| 2 \| 2.60 \| \| BIC/TAF/FTC,DRV/r \| 1 \| 1.30 \| \| DOR,DRV/c \| 1 \| 1.30 \| \| DRV/r,DTG/ABC/3TC \| 1 \| 1.30 \| \| DRV/r,DTG/RPV \| 1 \| 1.30 \| \| DTG BID,DRV/c \| 1 \| 1.30 \| \| DTG/ABC/3TC \| 1 \| 1.30 \| \| DTG/RPV \| 1 \| 1.30 \| \| I am unsure or unable to answer \| 1 \| 1.30 \| \| RPV,DTG/ABC/3TC \| 1 \| 1.30 \| \| TAF,DOR,DRV/c \| 1 \| 1.30 \| \| TDF/FTC,DRV/c \| 1 \| 1.30 \| \| Total \| 77 \| 100.00 \| | \|  \| Freq. \| Percent \| \| --- \| --- \| --- \| \| 1 PI,2 NRTI \| 24 \| 31.58 \| \| 1 PI,1 INSTI,2 NRTI \| 22 \| 28.95 \| \| 1 INSTI,2 NRTI \| 18 \| 23.68 \| \| 1 INSTI,1 NNRTI \| 5 \| 6.58 \| \| 1 PI,2 NRTI,1 NNRTI \| 2 \| 2.63 \| \| 1 INSTI,2 NRTI,1 NNRTI \| 1 \| 1.32 \| \| 1 PI,1 INSTI \| 1 \| 1.32 \| \| 1 PI,1 INSTI,1 NNRTI \| 1 \| 1.32 \| \| 1 PI,1 NNRTI \| 1 \| 1.32 \| \| 1 PI,1 NRTI,1 NNRTI \| 1 \| 1.32 \| \| Total \| 76 \| 100.00 \| |
| V7 | \|  \| Freq. \| Percent \| \| --- \| --- \| --- \| \| DOR,DTG BID,DRV/r BID \| 4 \| 6.45 \| \| DTG BID,DRV/r BID,FOS \| 4 \| 6.45 \| \| DOR,BIC/TAF/FTC \| 3 \| 4.84 \| \| I am unsure or unable to answer \| 3 \| 4.84 \| \| DOR,DTG BID,DRV/c \| 2 \| 3.23 \| \| DOR,DTG BID,FOS \| 2 \| 3.23 \| \| DOR,DTG,DRV/r \| 2 \| 3.23 \| \| DRV/r BID,BIC/TAF/FTC \| 2 \| 3.23 \| \| DTG BID,DRV/c/TAF/FTC \| 2 \| 3.23 \| \| DTG BID,DRV/r BID,FOS,TAF/FTC \| 2 \| 3.23 \| \| DTG BID,DRV/r BID,IBA \| 2 \| 3.23 \| \| DTG BID,DRV/r BID,TAF/FTC \| 2 \| 3.23 \| \| IBA,FOS \| 2 \| 3.23 \| \| BIC/TAF/FTC \| 1 \| 1.61 \| \| CAB,FOS,IBA \| 1 \| 1.61 \| \| DOR,BIC/TAF/FTC,DRV/c \| 1 \| 1.61 \| \| DOR,DRV/r BID,BIC/TAF/FTC \| 1 \| 1.61 \| \| DOR,DRV/r BID,FOS \| 1 \| 1.61 \| \| DOR,DTG BID,DRV/c/TAF/FTC \| 1 \| 1.61 \| \| DOR,DTG BID,DRV/r BID,FOS \| 1 \| 1.61 \| \| DOR,DTG BID,DRV/r BID,TAF/FTC \| 1 \| 1.61 \| \| DOR,DTG BID,FOS,TAF/FTC \| 1 \| 1.61 \| \| DOR,DTG,DRV/r BID \| 1 \| 1.61 \| \| DOR,DTG,DRV/r BID,IBA \| 1 \| 1.61 \| \| DOR,DTG,IBA \| 1 \| 1.61 \| \| DOR,FOS,BIC/TAF/FTC \| 1 \| 1.61 \| \| DOR,IBA,BIC/TAF/FTC \| 1 \| 1.61 \| \| DRV/r BID,IBA,FOS,TAF/FTC \| 1 \| 1.61 \| \| DRV/r BID,TDF/FTC,LEN \| 1 \| 1.61 \| \| DRV/r,DOR,FOS \| 1 \| 1.61 \| \| DTG BID,DOR/TDF/3TC \| 1 \| 1.61 \| \| DTG BID,DRV/r BID \| 1 \| 1.61 \| \| DTG BID,DRV/r BID,IBA,FOS \| 1 \| 1.61 \| \| DTG BID,DRV/r BID,TDF/FTC \| 1 \| 1.61 \| \| DTG BID,FOS \| 1 \| 1.61 \| \| DTG BID,FOS,TDF/FTC \| 1 \| 1.61 \| \| DTG,DRV/r BID \| 1 \| 1.61 \| \| DTG,DRV/r BID,FOS \| 1 \| 1.61 \| \| DTG,DRV/r BID,MVC \| 1 \| 1.61 \| \| FOS,BIC/TAF/FTC \| 1 \| 1.61 \| \| FOS,DTG,DRV/c \| 1 \| 1.61 \| \| FTC,TAF,ETR,FOS \| 1 \| 1.61 \| \| TAF,DOR,DTG BID,DRV/c \| 1 \| 1.61 \| \| Total \| 62 \| 100.00 \| | \|  \| Freq. \| Percent \| \| --- \| --- \| --- \| \| 1 PI,1 INSTI,1 EI \| 9 \| 15.25 \| \| 1 PI,1 INSTI,1 NNRTI \| 9 \| 15.25 \| \| 1 PI,1 INSTI,2 NRTI \| 7 \| 11.86 \| \| 1 INSTI,2 NRTI,1 NNRTI \| 4 \| 6.78 \| \| 1 PI,1 INSTI,2 NRTI,1 NNRTI \| 4 \| 6.78 \| \| 1 INSTI,1 EI,1 NNRTI \| 3 \| 5.08 \| \| 1 INSTI,1 EI,2 NRTI,1 NNRTI \| 3 \| 5.08 \| \| 1 INSTI,1 EI,2 NRTI \| 2 \| 3.39 \| \| 1 PI,1 EI,1 NNRTI \| 2 \| 3.39 \| \| 1 PI,1 INSTI \| 2 \| 3.39 \| \| 1 PI,1 INSTI,1 EI,1 NNRTI \| 2 \| 3.39 \| \| 1 PI,1 INSTI,1 EI,2 NRTI \| 2 \| 3.39 \| \| 2 EI \| 2 \| 3.39 \| \| 1 EI,2 NRTI,1 NNRTI \| 1 \| 1.69 \| \| 1 INSTI,1 EI \| 1 \| 1.69 \| \| 1 INSTI,2 EI \| 1 \| 1.69 \| \| 1 INSTI,2 NRTI \| 1 \| 1.69 \| \| 1 PI,1 EI,2 NRTI \| 1 \| 1.69 \| \| 1 PI,1 INSTI,1 NRTI,1 NNRTI \| 1 \| 1.69 \| \| 1 PI,1 INSTI,2 EI \| 1 \| 1.69 \| \| 1 PI,2 EI,2 NRTI \| 1 \| 1.69 \| \| Total \| 59 \| 100.00 \| |
| V8 | \|  \| Freq. \| Percent \| \| --- \| --- \| --- \| \| DTG BID,IBA,FOS \| 4 \| 6.56 \| \| DOR,DTG BID,DRV/r BID \| 3 \| 4.92 \| \| DOR,DTG BID,FOS \| 3 \| 4.92 \| \| I am unsure or unable to answer \| 3 \| 4.92 \| \| DOR,DTG BID,IBA \| 2 \| 3.28 \| \| DTG BID,DRV/r BID \| 2 \| 3.28 \| \| DTG BID,DRV/r BID,FOS \| 2 \| 3.28 \| \| DTG BID,DRV/r BID,TAF/FTC \| 2 \| 3.28 \| \| ETR,DTG BID,DRV/r BID \| 2 \| 3.28 \| \| FOS,BIC/TAF/FTC \| 2 \| 3.28 \| \| 3TC,DOR,ETR,DTG BID,DRV/r \| 1 \| 1.64 \| \| BIC/TAF/FTC,ATV/c \| 1 \| 1.64 \| \| CAB/RPV \| 1 \| 1.64 \| \| DOR,DRV/r BID,FOS \| 1 \| 1.64 \| \| DOR,DTG BID,ATV/c \| 1 \| 1.64 \| \| DOR,DTG BID,DRV/r \| 1 \| 1.64 \| \| DOR,DTG BID,DRV/r BID,FOS \| 1 \| 1.64 \| \| DOR,DTG BID,MVC \| 1 \| 1.64 \| \| DOR,DTG,ATV/c \| 1 \| 1.64 \| \| DOR,DTG,FOS \| 1 \| 1.64 \| \| DOR,DTG/RPV \| 1 \| 1.64 \| \| DOR,TAF/FTC,DRV/c \| 1 \| 1.64 \| \| DTG BID,DOR/TDF/3TC \| 1 \| 1.64 \| \| DTG BID,DRV/r \| 1 \| 1.64 \| \| DTG BID,DRV/r BID,IBA \| 1 \| 1.64 \| \| DTG BID,DRV/r BID,IBA,FOS \| 1 \| 1.64 \| \| DTG BID,DRV/r BID,TDF/FTC \| 1 \| 1.64 \| \| DTG BID,DRV/r,TAF/FTC \| 1 \| 1.64 \| \| DTG BID,FOS \| 1 \| 1.64 \| \| DTG BID,FOS,ATV/c \| 1 \| 1.64 \| \| DTG BID,FOS,DOR/TDF/3TC \| 1 \| 1.64 \| \| DTG BID,FOS,DRV/c \| 1 \| 1.64 \| \| DTG BID,FOS,LEN \| 1 \| 1.64 \| \| DTG BID,FOS,LEN,IBA \| 1 \| 1.64 \| \| DTG BID,FOS,TAF/FTC \| 1 \| 1.64 \| \| DTG BID,IBA,FOS,DOR/TDF/3TC \| 1 \| 1.64 \| \| DTG,DRV/c/TAF/FTC \| 1 \| 1.64 \| \| DTG,DRV/r BID,FOS \| 1 \| 1.64 \| \| ETR,DTG BID,DRV/r BID,FOS \| 1 \| 1.64 \| \| ETR,DTG BID,FOS \| 1 \| 1.64 \| \| ETR,DTG BID,IBA \| 1 \| 1.64 \| \| ETR,IBA,TAF/FTC \| 1 \| 1.64 \| \| FOS,DOR/TDF/3TC \| 1 \| 1.64 \| \| FOS,IBA,DTG \| 1 \| 1.64 \| \| IBA,FOS \| 1 \| 1.64 \| \| MVC,FOS,DRV/c/TAF/FTC \| 1 \| 1.64 \| \| Total \| 61 \| 100.00 \| | \|  \| Freq. \| Percent \| \| --- \| --- \| --- \| \| 1 INSTI,1 EI,1 NNRTI \| 9 \| 15.52 \| \| 1 PI,1 INSTI,1 NNRTI \| 8 \| 13.79 \| \| 1 INSTI,2 EI \| 6 \| 10.34 \| \| 1 PI,1 INSTI,1 EI \| 6 \| 10.34 \| \| 1 PI,1 INSTI,2 NRTI \| 6 \| 10.34 \| \| 1 INSTI,1 EI,2 NRTI \| 3 \| 5.17 \| \| 1 PI,1 INSTI \| 3 \| 5.17 \| \| 1 EI,2 NRTI,1 NNRTI \| 2 \| 3.45 \| \| 1 PI,1 INSTI,1 EI,1 NNRTI \| 2 \| 3.45 \| \| 1 INSTI,1 EI \| 1 \| 1.72 \| \| 1 INSTI,1 EI,2 NRTI,1 NNRTI \| 1 \| 1.72 \| \| 1 INSTI,1 NNRTI \| 1 \| 1.72 \| \| 1 INSTI,2 EI,2 NRTI,1 NNRTI \| 1 \| 1.72 \| \| 1 INSTI,2 NNRTI \| 1 \| 1.72 \| \| 1 INSTI,2 NRTI,1 NNRTI \| 1 \| 1.72 \| \| 1 INSTI,3 EI \| 1 \| 1.72 \| \| 1 PI,1 EI,1 NNRTI \| 1 \| 1.72 \| \| 1 PI,1 INSTI,1 NRTI,2 NNRTI \| 1 \| 1.72 \| \| 1 PI,1 INSTI,2 EI \| 1 \| 1.72 \| \| 1 PI,2 EI,2 NRTI \| 1 \| 1.72 \| \| 1 PI,2 NRTI,1 NNRTI \| 1 \| 1.72 \| \| 2 EI \| 1 \| 1.72 \| \| Total \| 58 \| 100.00 \| |
| V9 | \|  \| Freq. \| Percent \| \| --- \| --- \| --- \| \| I am unsure or unable to answer \| 7 \| 9.59 \| \| DTG BID,DRV/r BID,FOS \| 4 \| 5.48 \| \| DRV/r BID,IBA,FOS \| 3 \| 4.11 \| \| DTG BID,DRV/r BID,FOS,DOR/TDF/3TC \| 3 \| 4.11 \| \| DOR,DRV/r BID,FOS \| 2 \| 2.74 \| \| DOR,DTG BID,DRV/r BID,FOS \| 2 \| 2.74 \| \| DOR,DTG,DRV/c/TAF/FTC \| 2 \| 2.74 \| \| DTG BID,FOS,DRV/c/TAF/FTC \| 2 \| 2.74 \| \| IBA,FOS,DOR/TDF/3TC \| 2 \| 2.74 \| \| Other \| 2 \| 2.74 \| \| 3TC,DTG BID,DRV/r BID,FOS \| 1 \| 1.37 \| \| BIC/TAF/FTC,DRV/c \| 1 \| 1.37 \| \| CAB/RPV \| 1 \| 1.37 \| \| DOR,DRV/r BID,IBA \| 1 \| 1.37 \| \| DOR,DRV/r BID,IBA,FOS \| 1 \| 1.37 \| \| DOR,DRV/r BID,IBA,TAF/FTC \| 1 \| 1.37 \| \| DOR,DTG BID,DRV/r \| 1 \| 1.37 \| \| DOR,DTG BID,DRV/r BID,IBA,FOS \| 1 \| 1.37 \| \| DOR,DTG BID,IBA,FOS \| 1 \| 1.37 \| \| DOR,DTG BID,MVC,IBA \| 1 \| 1.37 \| \| DOR,DTG BID,TAF/FTC \| 1 \| 1.37 \| \| DOR,FOS,DRV/c/TAF/FTC \| 1 \| 1.37 \| \| DOR,FOS,LEN \| 1 \| 1.37 \| \| DOR,IBA,ABC/3TC \| 1 \| 1.37 \| \| DOR,IBA,FOS \| 1 \| 1.37 \| \| DOR,IBA,FOS,DRV/c \| 1 \| 1.37 \| \| DOR,IBA,FOS,TAF/FTC \| 1 \| 1.37 \| \| DOR/TDF/3TC \| 1 \| 1.37 \| \| DRV BID,DTG BID \| 1 \| 1.37 \| \| DRV/c/TAF/FTC \| 1 \| 1.37 \| \| DRV/r BID,FOS,DOR/TDF/3TC \| 1 \| 1.37 \| \| DRV/r BID,IBA,FOS,DOR/TDF/3TC \| 1 \| 1.37 \| \| DRV/r BID,IBA,FOS,TAF/FTC \| 1 \| 1.37 \| \| DRV/r,IBA,FOS,TDF/FTC \| 1 \| 1.37 \| \| DTG BID,DOR/TDF/3TC,DRV/c \| 1 \| 1.37 \| \| DTG BID,DRV/r BID,FOS,TAF/FTC \| 1 \| 1.37 \| \| DTG BID,DRV/r BID,IBA,FOS,DOR/TDF/3TC \| 1 \| 1.37 \| \| DTG BID,FOS,DOR/TDF/3TC \| 1 \| 1.37 \| \| DTG BID,FOS,RPV/TAF/FTC \| 1 \| 1.37 \| \| DTG BID,IBA,FOS,TAF/FTC \| 1 \| 1.37 \| \| ETR,DTG BID,DRV/c/TAF/FTC \| 1 \| 1.37 \| \| ETR,DTG BID,DRV/r BID,FOS,TAF/FTC \| 1 \| 1.37 \| \| ETR,DTG BID,DRV/r BID,TAF/FTC \| 1 \| 1.37 \| \| ETR,DTG BID,FOS \| 1 \| 1.37 \| \| ETR,DTG,DRV/r,TAF/FTC \| 1 \| 1.37 \| \| ETR,DTG,IBA,DRV/c/TAF/FTC \| 1 \| 1.37 \| \| IBA,FOS \| 1 \| 1.37 \| \| IBA,FOS,BIC/TAF/FTC \| 1 \| 1.37 \| \| IBA,FOS,CAB/RPV \| 1 \| 1.37 \| \| IBA,FOS,DTG/3TC \| 1 \| 1.37 \| \| IBA,FOS,TAF/FTC \| 1 \| 1.37 \| \| MVC,BIC/TAF/FTC \| 1 \| 1.37 \| \| TDF,DOR,DTG BID,DRV/r BID \| 1 \| 1.37 \| \| TDF,FOS,DTG/RPV,DRV/c \| 1 \| 1.37 \| \| Total \| 73 \| 100.00 \| | \|  \| Freq. \| Percent \| \| --- \| --- \| --- \| \| 1 PI,1 INSTI,2 NRTI,1 NNRTI \| 6 \| 9.38 \| \| 1 PI,1 INSTI,1 EI,2 NRTI,1 NNRTI \| 5 \| 7.81 \| \| 1 PI,1 INSTI,1 EI \| 4 \| 6.25 \| \| 1 INSTI,2 EI,1 NNRTI \| 3 \| 4.69 \| \| 1 PI,1 EI,1 NNRTI \| 3 \| 4.69 \| \| 1 PI,1 EI,2 NRTI,1 NNRTI \| 3 \| 4.69 \| \| 1 PI,1 INSTI,1 EI,2 NRTI \| 3 \| 4.69 \| \| 1 PI,2 EI \| 3 \| 4.69 \| \| 2 EI,2 NRTI,1 NNRTI \| 3 \| 4.69 \| \| 1 INSTI,1 EI,2 NRTI,1 NNRTI \| 2 \| 3.13 \| \| 1 INSTI,2 EI,2 NRTI \| 2 \| 3.13 \| \| 1 PI,1 INSTI,1 EI,1 NNRTI \| 2 \| 3.13 \| \| 1 PI,2 EI,1 NNRTI \| 2 \| 3.13 \| \| 1 PI,2 EI,2 NRTI \| 2 \| 3.13 \| \| 2 EI,1 NNRTI \| 2 \| 3.13 \| \| 1 EI,2 NRTI,1 NNRTI \| 1 \| 1.56 \| \| 1 INSTI,1 EI,1 NNRTI \| 1 \| 1.56 \| \| 1 INSTI,1 EI,2 NRTI \| 1 \| 1.56 \| \| 1 INSTI,1 NNRTI \| 1 \| 1.56 \| \| 1 INSTI,2 EI,1 NRTI \| 1 \| 1.56 \| \| 1 INSTI,2 NRTI,1 NNRTI \| 1 \| 1.56 \| \| 1 PI,1 INSTI \| 1 \| 1.56 \| \| 1 PI,1 INSTI,1 EI,1 NRTI \| 1 \| 1.56 \| \| 1 PI,1 INSTI,1 EI,1 NRTI,1 NNRTI \| 1 \| 1.56 \| \| 1 PI,1 INSTI,1 NNRTI \| 1 \| 1.56 \| \| 1 PI,1 INSTI,1 NRTI,1 NNRTI \| 1 \| 1.56 \| \| 1 PI,1 INSTI,2 EI,1 NNRTI \| 1 \| 1.56 \| \| 1 PI,1 INSTI,2 EI,2 NRTI,1 NNRTI \| 1 \| 1.56 \| \| 1 PI,1 INSTI,2 NRTI \| 1 \| 1.56 \| \| 1 PI,2 EI,2 NRTI,1 NNRTI \| 1 \| 1.56 \| \| 1 PI,2 NRTI \| 1 \| 1.56 \| \| 2 EI \| 1 \| 1.56 \| \| 2 EI,2 NRTI \| 1 \| 1.56 \| \| 2 NRTI,1 NNRTI \| 1 \| 1.56 \| \| Total \| 64 \| 100.00 \| |
| V10 | \|  \| Freq. \| Percent \| \| --- \| --- \| --- \| \| DRV/c/TAF/FTC \| 4 \| 7.69 \| \| DTG BID,DRV/c/TAF/FTC \| 4 \| 7.69 \| \| DTG BID,FOS,DRV/c \| 3 \| 5.77 \| \| FOS,DRV/c/TAF/FTC \| 3 \| 5.77 \| \| DRV/r BID,FOS,TAF/FTC \| 2 \| 3.85 \| \| DTG BID,DRV/c \| 2 \| 3.85 \| \| FOS,BIC/TAF/FTC \| 2 \| 3.85 \| \| FOS,TAF/FTC,DRV/c \| 2 \| 3.85 \| \| I am unsure or unable to answer \| 2 \| 3.85 \| \| IBA,FOS,DRV/c \| 2 \| 3.85 \| \| BIC/TAF/FTC \| 1 \| 1.92 \| \| BIC/TAF/FTC,DRV/c \| 1 \| 1.92 \| \| DOR,BIC/TAF/FTC \| 1 \| 1.92 \| \| DOR,DTG BID,FOS \| 1 \| 1.92 \| \| DOR,IBA,DRV/c/TAF/FTC \| 1 \| 1.92 \| \| DOR,IBA,FOS,TAF/FTC \| 1 \| 1.92 \| \| DRV/c,DTG BID \| 1 \| 1.92 \| \| DRV/c,FTV,TDF/FTC \| 1 \| 1.92 \| \| DRV/r BID,FOS \| 1 \| 1.92 \| \| DRV/r BID,IBA \| 1 \| 1.92 \| \| DRV/r BID,IBA,FOS \| 1 \| 1.92 \| \| DRV/r,IBA,FOS \| 1 \| 1.92 \| \| DRV/r,TAF/FTC \| 1 \| 1.92 \| \| DTG BID,DOR/TDF/3TC \| 1 \| 1.92 \| \| DTG BID,DOR/TDF/3TC,DRV/c \| 1 \| 1.92 \| \| DTG BID,DRV/r BID,DOR/TDF/3TC \| 1 \| 1.92 \| \| DTG BID,DRV/r,DOR/TDF/3TC \| 1 \| 1.92 \| \| DTG BID,DRV/r,FOS \| 1 \| 1.92 \| \| DTG BID,FOS,DRV/c/TAF/FTC \| 1 \| 1.92 \| \| DTG BID,FOS,TAF/FTC \| 1 \| 1.92 \| \| DTG BID,IBA,FOS,TAF/FTC \| 1 \| 1.92 \| \| FOS,DOR/TDF/3TC \| 1 \| 1.92 \| \| IBA,DRV/c/TAF/FTC \| 1 \| 1.92 \| \| IBA,FOS,DRV/c/TAF/FTC \| 1 \| 1.92 \| \| IBA,FOS,TDF/FTC \| 1 \| 1.92 \| \| TDF,DOR,DTG BID,DRV/r \| 1 \| 1.92 \| \| Total \| 52 \| 100.00 \| | \|  \| Freq. \| Percent \| \| --- \| --- \| --- \| \| 1 PI,1 EI,2 NRTI \| 8 \| 16.00 \| \| 1 PI,2 NRTI \| 6 \| 12.00 \| \| 1 PI,1 INSTI,2 NRTI \| 5 \| 10.00 \| \| 1 PI,1 INSTI,1 EI \| 4 \| 8.00 \| \| 1 PI,2 EI \| 4 \| 8.00 \| \| 1 INSTI,1 EI,2 NRTI \| 3 \| 6.00 \| \| 1 PI,1 INSTI \| 3 \| 6.00 \| \| 1 PI,1 INSTI,2 NRTI,1 NNRTI \| 3 \| 6.00 \| \| 1 INSTI,2 NRTI,1 NNRTI \| 2 \| 4.00 \| \| 1 PI,1 EI \| 2 \| 4.00 \| \| 1 EI,2 NRTI,1 NNRTI \| 1 \| 2.00 \| \| 1 INSTI,1 EI,1 NNRTI \| 1 \| 2.00 \| \| 1 INSTI,2 EI,2 NRTI \| 1 \| 2.00 \| \| 1 INSTI,2 NRTI \| 1 \| 2.00 \| \| 1 PI,1 EI,2 NRTI,1 NNRTI \| 1 \| 2.00 \| \| 1 PI,1 INSTI,1 EI,2 NRTI \| 1 \| 2.00 \| \| 1 PI,1 INSTI,1 NRTI,1 NNRTI \| 1 \| 2.00 \| \| 1 PI,2 EI,2 NRTI \| 1 \| 2.00 \| \| 2 EI,2 NRTI \| 1 \| 2.00 \| \| 2 EI,2 NRTI,1 NNRTI \| 1 \| 2.00 \| \| Total \| 50 \| 100.00 \| |
| V11 | \|  \| Freq. \| Percent \| \| --- \| --- \| --- \| \| DOR,DTG BID,FOS \| 5 \| 8.93 \| \| I am unsure or unable to answer \| 5 \| 8.93 \| \| DOR,DTG BID,MVC,FOS \| 4 \| 7.14 \| \| CAB/RPV \| 2 \| 3.57 \| \| DOR,DTG BID,MVC \| 2 \| 3.57 \| \| DOR,MVC,FOS \| 2 \| 3.57 \| \| DTG BID,DOR/TDF/3TC \| 2 \| 3.57 \| \| DTG BID,DRV/r BID,FOS \| 2 \| 3.57 \| \| DTG BID,DRV/r BID,MVC \| 2 \| 3.57 \| \| DTG BID,FOS,DOR/TDF/3TC \| 2 \| 3.57 \| \| DTG BID,MVC,FOS \| 2 \| 3.57 \| \| MVC,IBA,FOS \| 2 \| 3.57 \| \| BIC/TAF/FTC \| 1 \| 1.79 \| \| DOR,DTG BID,IBA \| 1 \| 1.79 \| \| DOR,DTG BID,IBA,FOS \| 1 \| 1.79 \| \| DOR,DTG BID,MVC,TAF/FTC \| 1 \| 1.79 \| \| DOR,DTG,IBA,DTG/RPV \| 1 \| 1.79 \| \| DOR,FOS,LEN \| 1 \| 1.79 \| \| DOR,IBA,FOS,BIC/TAF/FTC \| 1 \| 1.79 \| \| DOR,MVC \| 1 \| 1.79 \| \| DOR/TDF/3TC \| 1 \| 1.79 \| \| DRV/c/TAF/FTC \| 1 \| 1.79 \| \| DRV/r BID,MVC,FOS \| 1 \| 1.79 \| \| DTG BID,DOR,FOS,IBA \| 1 \| 1.79 \| \| DTG BID,DRV/r BID,IBA,TAF/FTC \| 1 \| 1.79 \| \| DTG BID,DRV/r BID,MVC,DOR/TDF/3TC \| 1 \| 1.79 \| \| DTG BID,DRV/r BID,MVC,FOS \| 1 \| 1.79 \| \| DTG BID,DRV/r BID,MVC,TDF/FTC \| 1 \| 1.79 \| \| DTG BID,DRV/r,FOS \| 1 \| 1.79 \| \| DTG BID,FOS,DRV/c/TAF/FTC \| 1 \| 1.79 \| \| DTG BID,FOS,TAF/FTC \| 1 \| 1.79 \| \| DTG BID,MVC,DOR/TDF/3TC \| 1 \| 1.79 \| \| ETR,DTG BID,MVC,FOS \| 1 \| 1.79 \| \| IBA,FOS,DOR/TDF/3TC \| 1 \| 1.79 \| \| MVC,DRV/c/TAF/FTC \| 1 \| 1.79 \| \| MVC,DTG/3TC,DTG/RPV \| 1 \| 1.79 \| \| Total \| 56 \| 100.00 \| | \|  \| Freq. \| Percent \| \| --- \| --- \| --- \| \| 1 INSTI,1 EI,1 NNRTI \| 8 \| 15.69 \| \| 1 INSTI,2 EI,1 NNRTI \| 7 \| 13.73 \| \| 1 PI,1 INSTI,1 EI \| 5 \| 9.80 \| \| 1 INSTI,1 EI,2 NRTI,1 NNRTI \| 4 \| 7.84 \| \| 1 PI,1 INSTI,1 EI,2 NRTI \| 3 \| 5.88 \| \| 2 EI,1 NNRTI \| 3 \| 5.88 \| \| 1 INSTI,1 NNRTI \| 2 \| 3.92 \| \| 1 INSTI,2 EI \| 2 \| 3.92 \| \| 1 INSTI,2 NRTI,1 NNRTI \| 2 \| 3.92 \| \| 3 EI \| 2 \| 3.92 \| \| 1 EI,1 NNRTI \| 1 \| 1.96 \| \| 1 INSTI,1 EI,1 NRTI,1 NNRTI \| 1 \| 1.96 \| \| 1 INSTI,1 EI,2 NNRTI \| 1 \| 1.96 \| \| 1 INSTI,1 EI,2 NRTI \| 1 \| 1.96 \| \| 1 INSTI,2 EI,2 NRTI,1 NNRTI \| 1 \| 1.96 \| \| 1 INSTI,2 NRTI \| 1 \| 1.96 \| \| 1 PI,1 EI,2 NRTI \| 1 \| 1.96 \| \| 1 PI,1 INSTI,1 EI,2 NRTI,1 NNRTI \| 1 \| 1.96 \| \| 1 PI,1 INSTI,2 EI \| 1 \| 1.96 \| \| 1 PI,2 EI \| 1 \| 1.96 \| \| 1 PI,2 NRTI \| 1 \| 1.96 \| \| 2 EI,2 NRTI,1 NNRTI \| 1 \| 1.96 \| \| 2 NRTI,1 NNRTI \| 1 \| 1.96 \| \| Total \| 51 \| 100.00 \| |
| S1 | \|  \| Freq. \| Percent \| \| --- \| --- \| --- \| \| BIC/TAF/FTC \| 41 \| 53.25 \| \| CAB/RPV \| 11 \| 14.29 \| \| DTG,DRV/c/TAF/FTC \| 9 \| 11.69 \| \| DTG/RPV \| 9 \| 11.69 \| \| DRV/c/TAF/FTC \| 3 \| 3.90 \| \| CAB/RPV,DRV/c \| 1 \| 1.30 \| \| DOR/TDF/3TC \| 1 \| 1.30 \| \| DTG,TAF/FTC \| 1 \| 1.30 \| \| I am unsure or unable to answer \| 1 \| 1.30 \| \| Total \| 77 \| 100.00 \| | \|  \| Freq. \| Percent \| \| --- \| --- \| --- \| \| 1 INSTI,2 NRTI \| 42 \| 55.26 \| \| 1 INSTI,1 NNRTI \| 20 \| 26.32 \| \| 1 PI,1 INSTI,2 NRTI \| 9 \| 11.84 \| \| 1 PI,2 NRTI, \| 3 \| 3.95 \| \| 1 PI,1 INSTI,1 NNRTI \| 1 \| 1.32 \| \| 2 NRTI,1 NNRTI \| 1 \| 1.32 \| \| Total \| 76 \| 100.00 \| |
| S2 | \|  \| Freq. \| Percent \| \| --- \| --- \| --- \| \| BIC/TAF/FTC \| 38 \| 53.52 \| \| EVG/c/TAF/FTC \| 16 \| 22.54 \| \| CAB/RPV \| 12 \| 16.90 \| \| DRV/c/TAF/FTC \| 1 \| 1.41 \| \| DTG,TAF/FTC \| 1 \| 1.41 \| \| DTG,TDF/FTC \| 1 \| 1.41 \| \| DTG/RPV \| 1 \| 1.41 \| \| EFV/TDF/FTC \| 1 \| 1.41 \| \| Total \| 71 \| 100.00 \| | \|  \| Freq. \| Percent \| \| --- \| --- \| --- \| \| 1 INSTI,2 NRTI \| 56 \| 78.87 \| \| 1 INSTI,1 NNRTI \| 13 \| 18.31 \| \| 1 PI,2 NRTI \| 1 \| 1.41 \| \| 2 NRTI,1 NNRTI \| 1 \| 1.41 \| \| Total \| 71 \| 100.00 \| |
| S3 | \|  \| Freq. \| Percent \| \| --- \| --- \| --- \| \| BIC/TAF/FTC \| 40 \| 70.18 \| \| CAB/RPV \| 14 \| 24.56 \| \| DTG,TAF/FTC \| 1 \| 1.75 \| \| DTG/3TC \| 1 \| 1.75 \| \| DTG/ABC/3TC \| 1 \| 1.75 \| \| Total \| 57 \| 100.00 \| | \|  \| Freq. \| Percent \| \| --- \| --- \| --- \| \| 1 INSTI,2 NRTI \| 42 \| 73.68 \| \| 1 INSTI,1 NNRTI \| 14 \| 24.56 \| \| 1 INSTI,1 NRTI \| 1 \| 1.75 \| \| Total \| 57 \| 100.00 \| |
| S4 | \|  \| Freq. \| Percent \| \| --- \| --- \| --- \| \| DTG/RPV \| 15 \| 27.78 \| \| DTG,TDF/FTC \| 14 \| 25.93 \| \| BIC/TAF/FTC \| 11 \| 20.37 \| \| DTG,DRV/c \| 3 \| 5.56 \| \| DOR,BIC/TAF/FTC \| 2 \| 3.70 \| \| CAB/RPV \| 1 \| 1.85 \| \| DOR,DTG \| 1 \| 1.85 \| \| DRV/c/TAF/FTC \| 1 \| 1.85 \| \| DTG BID,DRV/r BID,TAF/FTC \| 1 \| 1.85 \| \| DTG,TAF/FTC \| 1 \| 1.85 \| \| DTG/3TC,DRV/c \| 1 \| 1.85 \| \| DTG/ABC/3TC \| 1 \| 1.85 \| \| FTC,TDF,DTG \| 1 \| 1.85 \| \| TDF,DTG \| 1 \| 1.85 \| \| Total \| 54 \| 100.00 \| | \|  \| Freq. \| Percent \| \| --- \| --- \| --- \| \| 1 INSTI,2 NRTI, \| 28 \| 51.85 \| \| 1 INSTI,1 NNRTI, \| 17 \| 31.48 \| \| 1 PI,1 INSTI \| 3 \| 5.56 \| \| 1 INSTI,2 NRTI,1 NNRTI \| 2 \| 3.70 \| \| 1 INSTI,1 NRTI \| 1 \| 1.85 \| \| 1 PI,1 INSTI,1 NRTI \| 1 \| 1.85 \| \| 1 PI,1 INSTI,2 NRTI \| 1 \| 1.85 \| \| 1 PI,2 NRTI \| 1 \| 1.85 \| \| Total \| 54 \| 100.00 \| |
| S5 | \|  \| Freq. \| Percent \| \| --- \| --- \| --- \| \| CAB/RPV \| 30 \| 52.63 \| \| BIC/TAF/FTC \| 8 \| 14.04 \| \| DTG/RPV \| 7 \| 12.28 \| \| DRV/c/TAF/FTC \| 5 \| 8.77 \| \| DRV/r,TDF/FTC \| 2 \| 3.51 \| \| DTG,DOR/TDF/3TC \| 2 \| 3.51 \| \| DTG,DRV/c \| 2 \| 3.51 \| \| BIC/TAF/FTC,DRV/c \| 1 \| 1.75 \| \| Total \| 57 \| 100.00 \| | \|  \| Freq. \| Percent \| \| --- \| --- \| --- \| \| 1 INSTI,1 NNRTI \| 37 \| 64.91 \| \| 1 INSTI,2 NRTI \| 8 \| 14.04 \| \| 1 PI,2 NRTI \| 7 \| 12.28 \| \| 1 INSTI,2 NRTI,1 NNRTI \| 2 \| 3.51 \| \| 1 PI,1 INSTI \| 2 \| 3.51 \| \| 1 PI,1 INSTI,2 NRTI \| 1 \| 1.75 \| \| Total \| 57 \| 100.00 \| |
